# Single-Cell RNA-Seq Reveals Cellular Hierarchies and Impaired Developmental Trajectories in Pediatric Ependymoma

**DOI:** 10.1016/j.ccell.2020.06.004

**Published:** 2020-07-13

**Authors:** Johannes Gojo, Bernhard Englinger, Li Jiang, Jens M. Hübner, McKenzie L. Shaw, Olivia A. Hack, Sibylle Madlener, Dominik Kirchhofer, Ilon Liu, Jason Pyrdol, Volker Hovestadt, Emanuele Mazzola, Nathan D. Mathewson, Maria Trissal, Daniela Lötsch, Christian Dorfer, Christine Haberler, Angela Halfmann, Lisa Mayr, Andreas Peyrl, Rene Geyeregger, Benjamin Schwalm, Monica Mauermann, Kristian W. Pajtler, Till Milde, Marni E. Shore, Jack E. Geduldig, Kristine Pelton, Thomas Czech, Orr Ashenberg, Kai W. Wucherpfennig, Orit Rozenblatt-Rosen, Sanda Alexandrescu, Keith L. Ligon, Stefan M. Pfister, Aviv Regev, Irene Slavc, Walter Berger, Mario L. Suvà, Marcel Kool, Mariella G. Filbin

**Affiliations:** 1 Department of Pediatric Oncology, Dana-Farber Boston Children's Cancer and Blood Disorders Center, Boston, MA 02215, USA; 2 Department of Pediatrics and Adolescent Medicine, Comprehensive Center for Pediatrics, Medical University of Vienna, 1090 Vienna, Austria; 3 Broad Institute of Harvard and MIT, Cambridge, MA 02142, USA; 4 Hopp Children's Cancer Center (KiTZ), 69120 Heidelberg, Germany; 5 Division of Pediatric Neurooncology, German Cancer Research Center (DKFZ), 69120 Heidelberg, Germany; 6 Institute of Cancer Research, Comprehensive Cancer Center, Medical University of Vienna, 1090 Vienna, Austria; 7 Department of Cancer Immunology and Virology, Dana-Farber Cancer Institute and Harvard Medical School, Boston, MA 02215, USA; 8 Department of Pathology and Center for Cancer Research, Massachusetts General Hospital and Harvard Medical School, Boston, MA 02114, USA; 9 Department of Data Sciences, Dana-Farber Cancer Institute, Boston, MA 02215, USA; 10 Department of Neurosurgery, Medical University of Vienna, 1090 Vienna, Austria; 11 Division of Neuropathology and Neurochemistry, Department of Neurology, Medical University of Vienna, 1090 Vienna, Austria; 12 Clinical Cell Biology, Children's Cancer Research Institute (CCRI), 1090 Vienna, Austria; 13 Department of Paediatric Haematology and Oncology, Heidelberg University Hospital, 69120 Heidelberg, Germany; 14 Department of Oncologic Pathology, Dana-Farber Cancer Institute, Boston, MA 02215, USA; 15 Department of Pathology, Boston Children's Hospital, Boston, MA 02115, USA; 16 Department of Pathology, Brigham and Women's Hospital, Boston Children's Hospital, Dana-Farber Cancer Institute, Boston, MA 02215, USA; 17 Klarman Cell Observatory, Broad Institute of MIT and Harvard, Cambridge, MA 02142, USA; 18 Howard Hughes Medical Institute, Department of Biology, Massachusetts Institute of Technology, Cambridge, MA 02140, USA; 19 Princess Máxima Center for Pediatric Oncology, 3584 CS Utrecht, the Netherlands

**Keywords:** Pediatric ependymoma, EPN, single-cell RNA sequencing, intratumoral heterogeneity, cellular hierarchy, aberrant development, glial development, differentiation trajectory, cancer stem cells, target identification

## Abstract

Ependymoma is a heterogeneous entity of central nervous system tumors with well-established molecular groups. Here, we apply single-cell RNA sequencing to analyze ependymomas across molecular groups and anatomic locations to investigate their intratumoral heterogeneity and developmental origins. Ependymomas are composed of a cellular hierarchy initiating from undifferentiated populations, which undergo impaired differentiation toward three lineages of neuronal-glial fate specification. While prognostically favorable groups of ependymoma predominantly harbor differentiated cells, aggressive groups are enriched for undifferentiated cell populations. The delineated transcriptomic signatures correlate with patient survival and define molecular dependencies for targeted treatment approaches. Taken together, our analyses reveal a developmental hierarchy underlying ependymomas relevant to biological and clinical behavior.

## Highlights

• Cellular hierarchies in ependymoma reflect impaired neurodevelopment
• Ependymoma cells follow neuronal-, astrocytic-, and ependymal-like trajectories
• Undifferentiated programs confer inferior prognosis and are enriched at recurrence
• Preclinical data suggest feasibility of inhibiting undifferentiated subpopulations

## Graphical Abstract

**Figure:**
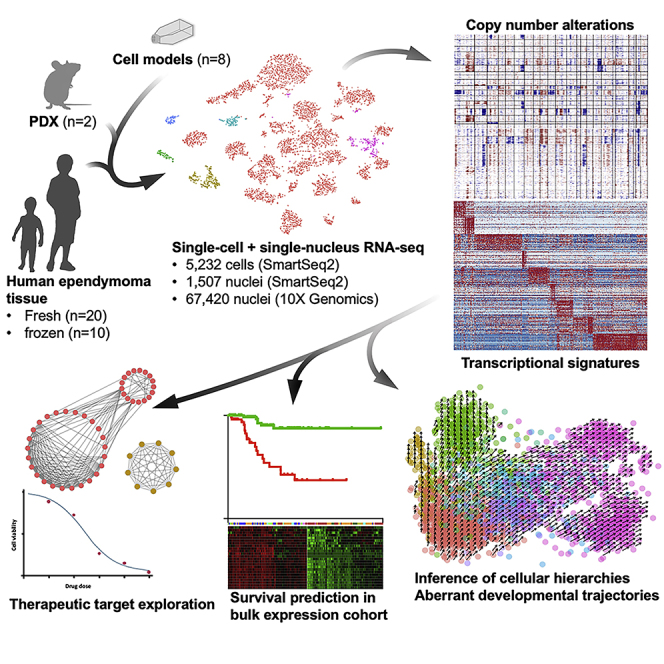

## Teaser

Gojo et al. use single-cell RNA sequencing to investigate intratumoral heterogeneity and cellular hierarchy in pediatric ependymoma, identifying impaired neurodevelopmental trajectories. The malignant trajectories reveal therapeutic targets and prognostic signatures in ependymoma.

## Significance

**Despite extensively growing knowledge on the molecular biology of ependymoma, effective treatments are still lacking for aggressive subtypes. By applying single-cell RNA sequencing, we comprehensively identify the cellular hierarchy within ependymoma spanning undifferentiated and differentiated tumor cell states across all major molecular groups. This not only refines the concept of these established molecular groups but also provides a biological context for the well-known capacity of aggressive ependymomas for late recurrence and treatment resistance. The transcriptomic signatures presented here also are prognostic within established high-risk ependymoma groups and provide druggable targets for currently approved compounds. Consequently, the characterized cell states could serve as promising future therapeutic targets and biomarkers for clinical trials.**

## Introduction

Ependymomas (EPNs) are central nervous system (CNS) tumors encompassing highly aggressive as well as more benign tumors (Pajtler et al., 2015). The discovery of nine main molecular groups based on genome-wide DNA-methylation patterns has facilitated more precise diagnosis and risk stratification of EPN patients (Pajtler et al., 2015, 2017, 2018; Ramaswamy et al., 2016; Cavalli et al., 2018). Posterior fossa (PF) group A (PF-A) EPN, characterized by H3K27 hypomethylation, overexpression of *EZHIP*, or infrequent H3K27M mutations (Panwalkar et al., 2017; Hubner et al., 2019; Pajtler et al., 2018), and supratentorial (ST) EPN with *C11orf95-RELA*-fusions (ST-RELA) have been identified as the most aggressive molecular groups, occurring predominantly in younger patients. In contrast, PF group B (PF-B), ST-EPN with *YAP1*-fusions (ST-YAP1), and most spinal EPNs exhibit a favorable prognosis (Pajtler et al., 2015; Parker et al., 2014).

Cross-species genomic analyses have suggested deregulated, regionally specific neural stem/radial glial cells in the developing brain as putative EPN cells of origin (Johnson et al., 2010; Mohankumar et al., 2015; Taylor et al., 2005). However, developmental origins and candidate driver genes of EPN have been largely informed by mouse models or bulk RNA-sequencing (RNA-seq) analyses, or were conducted in limited subsets of EPN groups (Vladoiu et al., 2019; Pajtler et al., 2015; Taylor et al., 2005; Mohankumar et al., 2015). We therefore hypothesized that we could unravel distinct intratumoral subpopulations and resolve the cellular programs orchestrating key proliferation, “stemness", and chemoresistance traits in EPN by using integrative single-cell transcriptomics of patients' primary and recurrent tumors.

## Results

### scRNA-Seq Profiling of Fresh and Frozen Patient EPN Tissue and Corresponding Tumor Models Identifies Malignant and Non-malignant Cells

**Figure 1:**
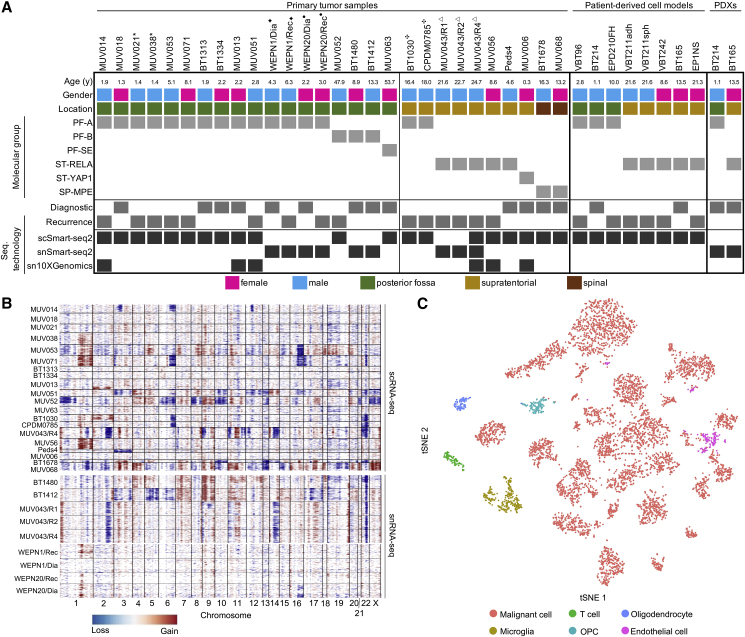
Classification of Human EPN Single-Cell Transcriptomes (A) Clinical and molecular details of the human EPN dataset of fresh/frozen patient samples (n = 28), patient-derived cell models (n = 8), and PDXs (n = 2). scRNA-seq technologies applied per sample are indicated. Matched pairs (n = 5) are indicated by superscript symbols. (B) Inference of copy-number alterations (CNAs) from scRNA-seq (top) and snRNA-seq (bottom) data on the basis of average relative expression of sliding windows of 100 genes. Each row corresponds to a cell, ordered by tumor and clustered within each tumor by CNA patterns. (C) t-Distributed stochastic neighbor embedding (tSNE) of all cells derived from scRNA-seq and snRNA-seq. Cells are colored according to presence of CNAs and similarity to expression signatures of non-malignant cell populations (T cells, oligodendroglial precursor cells [OPC], oligodendrocytes, microglia, endothelial cells). See also Table S1.

We aimed at generating single-cell transcriptomic data from EPN tissue comprising all major molecular groups and anatomical locations. We analyzed 20 fresh surgical tumor specimens from 18 EPN patients, eight patient-derived cell models, and two patient-derived xenograft (PDX) models by full-length transcriptome single-cell RNA-seq (scRNA-seq; Smart-seq2 [Picelli, 2019]) (Figure 1A and Table S1). Four of the cell models were matched to fresh patient tumors (Table S1). Additionally, snap-frozen EPN tissues (n = 14) were included for single-nucleus RNA-seq (snRNA-seq) analysis (Smart-seq2 and/or 10X Genomics [Habib et al., 2016]) (Figure 1A and Table S1). We performed molecular group analysis by DNA methylation on each sample (Figure 1A and Table S1). In total, 74,927 single tumor cells/nuclei were analyzed: 5,232 cells profiled by scRNA-seq passed quality control, with a median of 4,652 genes detected per cell. snRNA-seq yielded an additional 2,137/67,420 nuclei (Smart-seq2/10X Genomics), with a median of 3,072/3,089 genes detected per nucleus, respectively.

To classify cells as malignant or non-malignant, we inferred genome-wide copy-number alterations (CNAs) from scRNA-seq/snRNA-seq data (Tirosh et al., 2016) (Figure 1B) and compared CNA profiles with those of non-malignant controls (Tirosh et al., 2016; Filbin et al., 2018). Large-scale CNAs were detected in the majority of samples (n = 24/28) and matched those derived independently from DNA-methylation data. CNAs included the hallmark chromosome (Chr) 1q gain (PF-A, ST-RELA) and other typical EPN-CNAs including Chr6q deletion (PF-A, PF-B, ST-Midline), monosomy of Chr22 (all groups), and Chr5p gain (PF-A and PF-B) (Pajtler et al., 2015). Next, we clustered cells across all samples according to their transcriptional profiles (Figure 1C). Several cell clusters lacked CNA and showed high expression levels of marker genes specific to normal cell types, including microglia (n = 296, e.g., *CD14*, *FCER1G*, *CSF1R*), T cells (n = 111, e.g., *CD3E*, *CD4*, *CD8A*), oligodendrocyte precursor cells (OPCs) (n = 101, e.g., *OLIG1*, *APOD*, *PDGFRA*), oligodendrocytes (n = 149, e.g., *MBP*, *PLP1*, *MOG*), and endothelial cells (n = 153, *IFITM1*, *CAV1*, *TM4SF1*). Those cells were considered non-malignant and excluded from downstream analyses.

Thus, the two approaches concordantly segregated cells into malignant and normal subsets, overall classifying 87.8% of cells across all tumor samples as malignant by Smart-seq2.

### Posterior Fossa EPN Is Composed of Multiple Differentiated as well as Undifferentiated Tumor Cell Types

**Figure 2:**
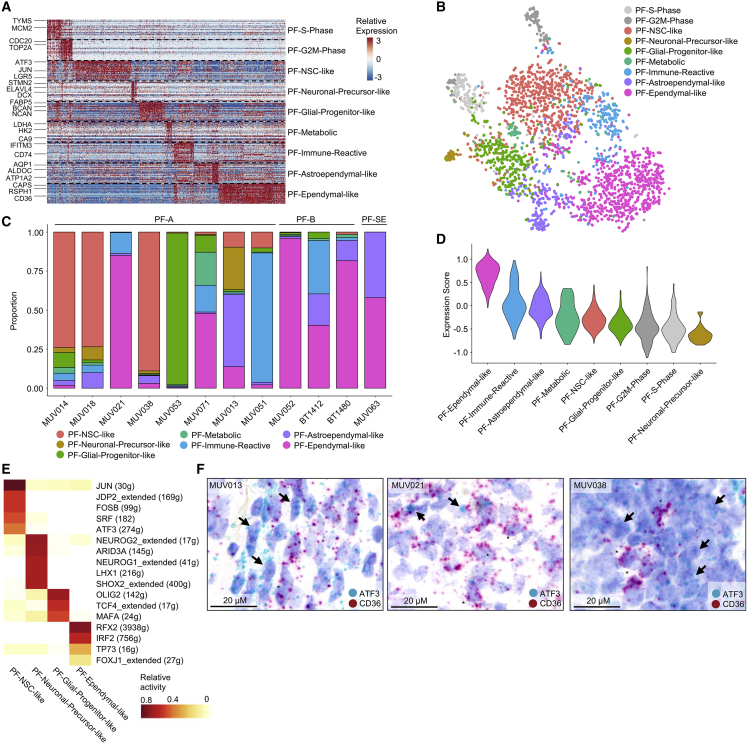
Intratumoral Heterogeneity in PF-EPN (A) Relative expression (color bar) across 2,772 malignant cells (columns) of the top 30 genes (rows) for each PF metaprogram. (B) tSNE plot of all fresh PF tumor cells, colored on the basis of assigned PF metaprogram. (C) Relative frequency of each metaprogram per sample, shown for PF-A, PF-B, and PF-SE. MUV021 versus MUV038: PF-Ependymal-like cells (p = 2.9 × 10^−112^, Fisher's exact test); PF-NSC-like cells (p = 3.1 × 10^−123^, Fisher's exact test). (D) Scoring of FOXJ1 target gene (Stauber et al., 2017) expression across PF-EPN metaprograms. (E) Average relative activity of PF-EPN subpopulation-specific TF regulons by SCENIC. (F) RNA *in situ* hybridization of PF-NSC-like (*ATF3*) and PF-Ependymal-like (*CD36*) markers in formalin-fixed, paraffin-embedded (FFPE) tissue sections matched to scRNA-seq samples. Arrows and asterisks indicate representative cells positive for *ATF3* or *CD36*, respectively. The scale bars indicate 20 μm. See also Figure S1 and Table S2. Metaprogram Genes and Assignments, Related to Figures 2 and 4, Table S3. PF-EPN and ST-EPN Signatures, Related to Figures 2 and 4, Table S4. GO Term Enrichment Using Cell Population-Specific Signature and Top 30 Metaprogram Genes, Related to Figures 2 and 4, Table S5. Relative TF Activity and Metaprogram-Specific TFs, Related to Figures 2 and 4.

To characterize posterior fossa EPN (PF-EPN), we used 14 PF patient samples as primary cohort and used an additional four samples (WEPN1/Dia, WEPN1/Rec, WEPN20/Dia, WEPN20/Rec) for validation and investigation of tumor evolution at recurrence (Figure 1A). Two samples (BT1313 and BT1334) mostly included immune cells and thus were excluded from non-negative matrix factorization (NMF) analysis. We identified recurrent transcriptional programs by NMF and merged them into nine metaprograms (Figures 2A, S1A, and S1B; Tables S2 and S3). Moreover, we applied graph-based clustering as an independent approach. Both methods yielded highly congruent clustering, corroborating final gene signatures that define cell subpopulations (Figures S1C and S1D). We examined gene signatures for known cell-type-specific marker genes, integrated them with human and mouse reference atlas datasets of developmental and adult brain cell types (La Manno et al., 2016; Nowakowski et al., 2017; Zeisel et al., 2018), and functionally annotated them using Gene Ontology (GO) term enrichment (Raudvere et al., 2019) (Figure S1E and Table S4). Two metaprograms were strongly associated with cell-cycle genes and were consequently termed PF-S-Phase (e.g., *TYMS*, *PCNA*, *MCM2/4/5/7*) and PF-G2M-Phase (e.g., *CDC20*, *CCNB1*, *PLK1*) (Table S4). We interpreted cells scoring highly for these metaprograms as populations of cycling cells, which almost exclusively occurred in PF-A samples (Figure S1F). Two additional metaprograms closely resembled more mature cell types: PF-Ependymal-like cells expressed ciliogenesis markers (e.g., *DNAAF1*, *DNAI2*, *RSPH1*) and shared global transcriptional programs with differentiated ependymal cell types in murine reference datasets (Table S4). PF-Astroependymal signature contained canonical marker genes for astrocytes (e.g., *AQP4*, *ALDOC*, *S100B*, *GFAP*) and strongly correlated with astrocytes in both human and mouse reference atlases. Three additional metaprograms resembled immature stem-like cells and neuronal or glial lineage precursors. The PF-Neural-Stem-Cell-like program (PF-NSC-like) was associated with transcriptional activity and stemness (e.g., *FOS*, *LGR5*, *ZFP36*, *EGR1*, *JUN*). This program depicts a broadly immature cell type that might be further refined in the future as more normal cell atlases become available. PF-Neuronal-Precursor-like cells were characterized by genes involved in neuronal fate (*STMN1/2/4*, *SOX4/11*, *ELAVL4*, *L1CAM*), while the PF-Glial-Progenitor-like program was linked to early glial lineage determination (*FABP5*, *BCAN*). Two more metaprograms were identified that reflected distinct cellular metabolic/immune-reactive states and contained genes that were strongly associated with glycolytic (PF-Metabolic, e.g., *PGK1*, *GAPDH*, *PFKP*) and immune-effector processes (PF-Immune-Reactive, e.g., *IFITM3*, *HLA-C*, *C4B*). Within the PF-Metabolic program, several enriched GO terms also indicated hypoxia response (Table S4).

To further validate the robustness of our metaprograms, we generated small nuclear RNA-seq (snRNA-seq) data by an alternative scRNA-seq technology (10X Genomics) from three frozen PF-A samples matching analyzed fresh samples and uncovered similar gene programs (Figure S1G). Furthermore, metaprograms were also partially recapitulated in PF-A PDXs and *in vitro* cell models. However, PDX models most closely resembled primitive cell states identified from fresh patient tissue (Figure S1H).

We next compared scRNA-seq profiles among all PF tumors (Figures 2A and 2B). We found that the more aggressive molecular group of PF-A tumors encompassed a high complexity of metaprograms per tumor and was enriched for less differentiated cell states (p < 0.001, Fisher's exact test) (Figures 2C and S1I). Interestingly, proliferating cells were restricted to the three undifferentiated PF-NSC-like, PF-Neuronal-Precursor-like, and PF-Glial-Progenitor-like subpopulations (Figure S1J). In contrast, samples of the more benign molecular groups PF-B and PF-subependymoma (PF-SE) were exclusively composed of less proliferative, more differentiated cell populations expressing PF-Ependymal-like and PF-Astroependymal-like programs (Figures 2C, S1I, and S1J).

We next investigated potential transcription factors (TFs) regulating these programs. We found FOXJ1 target genes (Stauber et al., 2017) to be preferentially expressed in the PF-Ependymal-like subpopulation of cells (p < 0.001, Wilcoxon’s rank-sum test), which is as such not exclusive to PF-B but rather a marker of ciliogenic programs (Mack et al., 2018), and ependymal differentiation was also observed in a subset of PF-A tumors (Figures 2C, 2D, and S1K).

In addition, we comprehensively inferred TF regulatory networks by single-cell regulatory network inference and clustering (SCENIC) analysis (Aibar et al., 2017) (Table S5). More than half of the highly active TF regulons identified in our dataset (shared and subtype-specific) had also been described in a previous study (Mack et al., 2018). In addition, SCENIC suggested additional TF regulons within the PF-Ependymal-like metaprogram, including *RFX2* and *TP73*, both of which are implicated in ciliogenesis (Figure 2E) (Nemajerova et al., 2016; Wildung et al., 2019; Chung et al., 2014). TF signatures of PF-NSC-like cells included *JUN*, *FOS*, and *ATF3*, all of which are also top signature genes of this subpopulation (Figure 2E). Other TF regulons with high activity in PF-NSC-like cells were *SRF* and *JDP2*, both of which are implicated in repression of cell differentiation and pluripotency induction (Figure 2E) (Wang et al., 2019a; Ikeda et al., 2018). The PF-Neuronal-Precursor-like program also showed a selective TF signature including *NEUROG1/2* and *ARID3A* (Figure 2E), described to regulate neurogenesis (Han et al., 2018) and promote oncogenic stemness (Dausinas et al., 2020). Lastly, PF-Glial-Progenitor-like cells exhibited TF signatures including *OLIG2*, compatible with early glial fate determination, supporting the progenitor-like state of this metaprogram (Chen et al., 2012) (Figure 2E).

We next aimed at confirming metaprogram expression in intact tumor tissue by using RNA *in situ* hybridization (RNA-ISH) in PF-A samples. In PF-A tumor slides, analysis of top signature genes demonstrated mutually exclusive expression of PF-Ependymal-like (*CD36*) and PF-NSC-like (*ATF3*) markers, and some extent of spatial clustering of cells expressing the corresponding programs (Figure 2F). Validating scRNA-seq data on our matched tumor pair, we also found an increase of cells expressing the PF-NSC-like signature and reduced expression of the PF-Ependymal-like program in metastatic recurrent PF-A tumor tissue MUV038 as compared with its matched preceding local recurrence MUV021 by RNA-ISH (Figure 2F).

Taken together, scRNA-seq analysis of PF-EPN cells reveals diverse tumor cell subpopulations driven by specific TF regulatory circuits. Undifferentiated NSC-like and early neuronal-precursor-like tumor cell types are enriched in aggressive PF-A tumors, whereas more differentiated, ependymal-like cell types are predominantly observed in favorable prognostic group PF-B and PF-SE tumors.

### Malignant Differentiation Trajectories Have Prognostic Impact and Can Be Therapeutically Targeted

**Figure 3:**
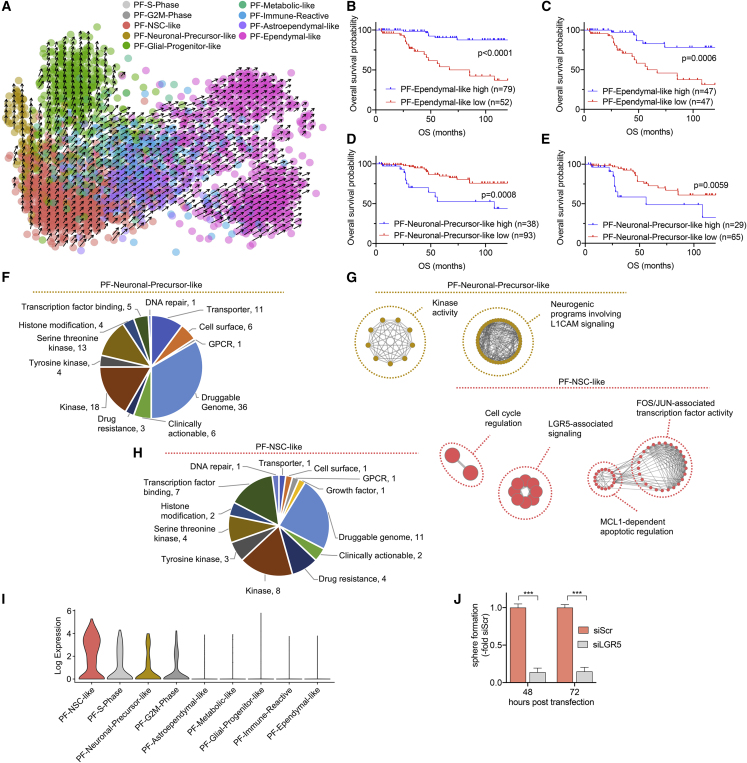
Malignant Cell Differentiation Trajectories and Their Prognostic and Therapeutic Relevance in PF-EPN (A) RNA velocity estimate of PF metaprograms. Each dot represents a cell. Cells are colored according to corresponding metaprograms. Arrows represent velocity that estimates extrapolated future cell states. (B and C) Overall survival (OS) stratification of PF (B) and PF-A (C) EPN according to high or low relative expressions of top 30 genes for PF-Ependymal-like metaprogram in bulk mRNA expression data. Significance levels were determined by log-rank test. (D and E) Overall survival (OS) stratification of PF (D) and PF-A (E) EPN tumors according to high or low relative expression of top 30 genes for PF-Neuronal-Precursor-like metaprogram in bulk mRNA expression data. Significance levels were determined by log-rank test. (F) Pie chart of candidate genes for therapeutic targeting, analyzed by integrating signature genes of PF-Neuronal-Precursor-like metaprogram with DGIdb. Gene hits in the “Druggable genome” and “Clinically actionable” references are shown. (G) Network maps of significantly enriched GO terms (p = 0.01, g:SCS Threshold) derived from G-Profiler pathway enrichment analysis of DGIdb gene hits for PF-Neuronal-Precursor-like and PF-NSC-like metaprograms. (H) Pie chart of candidate genes for therapeutic targeting, analyzed by integrating signature genes of PF-NSC-like metaprogram with DGIdb. Gene hits in the “Druggable genome” and “Clinically actionable” references are shown. (I) Log-transformed expression of LGR5 across PF-EPN metaprograms. (J) Relative sphere area at 48 h and 72 h post transfection of the PF-EPN cell model VBT96 with siLGR5 or non-targeting siRNA (siScr). Sphere formation upon siLGR5 KD is depicted relative to respective siScr controls. ^∗∗∗^p < 0.001, two-tailed Student's t test. Data are presented as mean ± SEM of triplicate values. See also Figures S2 and S3; Tables S6 and S7.

We next sought to dissect potential cellular differentiation trajectories of PF-EPN cancer cells by RNA velocity (La Manno et al., 2018). Our PF scRNA-seq dataset revealed a cellular hierarchy that was initiated in PF-NSC-like cells and followed three main trajectories: The majority of cells differentiated along the astroependymal lineage toward an ependymal-like cell type (Figure 3A). A second axis occurred toward glial progenitor-like cells, which expressed genes characteristic for certain radial glial cells and early glial lineage markers (Miller et al., 2019). A third but minor cell population was composed of PF-Neuronal-Precursor-like cells expressing genes of early neuronal fate. This putative developmental trajectory was consistent across all PF tumors (Figure S2A).

We next evaluated the prognostic impact of transcriptional signatures in an extensive bulk RNA expression cohort of 131 PF-EPN cases annotated for survival. Segregation by high versus low PF-Ependymal-like signature revealed that tumors expressing high levels of this program were either PF-B or a subset of PF-A with distinctly favorable clinical outcome (Figures 3B, S2B, and S2C). In support of this finding, the PF-Ependymal-like signature significantly stratified patient overall survival (OS) and progression-free survival (PFS) across all PF tumors, but also within PF-A patients alone (Figures 3C, S2D, and S2E). This was supported by multivariate Cox regression analysis confirming the PF-Ependymal-like signature as an independent predictor of both OS and PFS (Table S6). Moreover, PF-A cases with low levels of the PF-Ependymal-like signature exhibited a 7.3-fold increased risk of death (hazard ratio 7.3, p < 0.05, Table S6). With respect to the PF-Neuronal-Precursor-like signature, PF-B tumors clustered exclusively to the “low” cohort, while PF-A tumors again segregated into both “high” and “low” clusters (Figure S2F). EPNs with high expression levels for this PF-Neuronal-Precursor-like program conferred significantly worse OS compared with tumors with a low score, even when including only PF-A patients (Figures 3D, 3E, S2F, and S2G). Conversely, all remaining programs, despite clustering patient cohorts into “high” and “low” expressing populations, did not correlate with patient outcome (data not shown).

In addition, we found a positive correlation between ependymal differentiation and higher age of EPN patients (Figure S3A), while an inverse correlation was evident between PF-Neuronal-Precursor-specific/PF-NSC-specific markers and age (Figures S3B and S3C).

We further tested whether tumor samples with 1q-gain, a well-described predictor of poor outcome (Gojo et al., 2017; Pajtler et al., 2017), are enriched for undifferentiated programs. While we observed a trend toward lower numbers of differentiated PF-Astroependymal-like cells and increased amounts of stem-like PF-Glial-Progenitor-like cells in 1q-gained samples, the power of this analysis was limited due to small sample size (n = 7) and high interpatient heterogeneity (Figure S3D).

As tumor recurrence represents the major clinical problem in EPN management, we also investigated three matched PF-A pairs (one tumor pair in the discovery cohort, two additional tumor pairs in the validation cohort) of diagnostic/early-recurrence versus late-recurrence samples. We observed a shift from predominantly differentiated PF-Ependymal-like and PF-Astroependymal cells at diagnosis to undifferentiated PF-NSC-like, PF-Glial-Progenitor-like, and proliferating cells at recurrence (Figure S3E).

To inform improved future therapeutic approaches, we investigated potentially targetable pathways and biomarkers specific to the identified expression signatures. We integrated cell-population-specific genes with the Drug Gene Interaction database (DGIdb) (Cotto et al., 2018), prioritizing for target genes for which pharmacological intervention might be available. For the PF-Neuronal-Precursor-like subpopulation, these analyses indicated druggable vulnerabilities including the epigenetic regulators *HDAC2*, *DNMT3A*, and *BRD3*, signaling-associated genes *PIK3R3*, *MAP4K4*, and *MAPK6*, microtubule-associated genes *TUBA1A*, *TUBB*, *TUBB2A*, *TUBB2B*, and *TUBB3*, and activin receptor genes *ACVR2A* and *ACVR2B* (Figure 3F and Table S7). PF-Neuronal-Precursor-like cells also expressed *ABCC5*, a poorly described ABC transporter expressed at the blood-brain barrier with the ability to export several anticancer agents (Assaraf, 2006). Functional network maps of significantly enriched GO terms derived from DGIdb hits of the PF-Neuronal-Precursor-like program showed enrichment of kinase activity-related mechanisms as well as *L1CAM*-orchestrated signaling implicated in neuronal development (Figure 3G and Table S7). As a first proof of principle for functional validation, we inhibited HDAC2 by panobinostat in two PF-A cell models expressing this gene (Figure S3F). Indeed, we observed a significant reduction of cell viability (Figure S3G) as well as inhibition of secondary sphere formation (Figure S3H).

Hits for the PF-NSC-like program included Wnt-signaling regulator *LGR5* and the anti-apoptotic gene *MCL1*, as well as the cancer stemness-associated genes as potential druggable vulnerabilities (Xu et al., 2019) (Figure 3H and Table S7). Functional network maps of the PF-NSC-like subpopulation supported these findings and equally showed enrichment for pathways converging on LGR5 signaling, FOS/FOSB/JUN/JUNB-mediated transcription factor activity, and MCL1-dependent antiapoptotic mechanisms, as well as cell-cycle regulation, likely reflecting a high proportion of actively proliferating cells within this subpopulation (Figure 3G and Table S7). Indeed, *LGR5* was significantly upregulated in PF-NSC-like cells (Figure 3I), and small interfering RNA (siRNA)-mediated *LGR5* knockdown (KD) experiments significantly inhibited sphere formation in the *LGR5*-expressing patient-derived PF-A model, VBT96 (Figures 3J and S3I).

We conclude that single-cell hierarchies in PF-EPN reflect physiologically stalled differentiation trajectories, with cells ranging from NSC-like tumor cell populations to neuronal progenitor-like, immature glial-like, and more differentiated ependymal-like lineages. These transcriptional states serve as robust risk prognosticators beyond established molecular groups and suggest the first subpopulation-specific, targetable molecular tumor dependencies for future therapeutic strategies.

### ST-EPN Is Composed of Multiple, Molecular Group-Specific Cancer Cell Types

**Figure 4:**
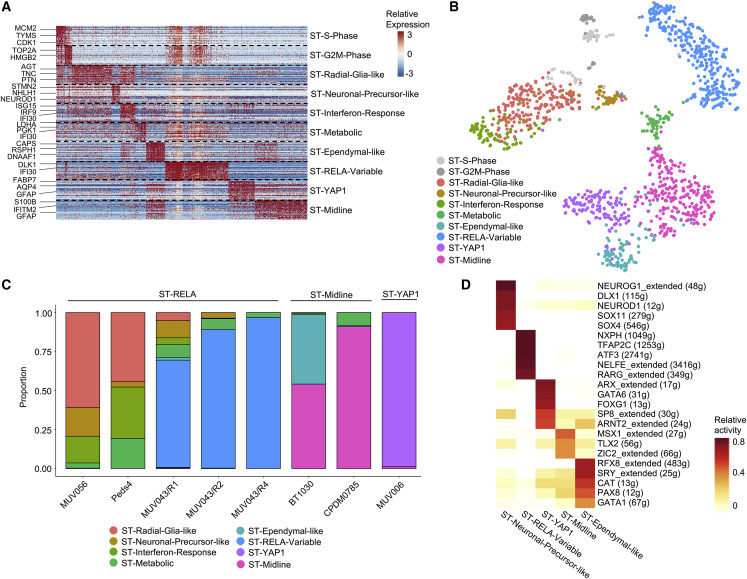
Malignant Transcriptional Programs in ST-EPN (A) Relative expression (color bar) across 1,296 malignant cells (columns) of the top 30 genes (rows) for each metaprogram. (B) tSNE plot of all fresh ST tumor cells, colored on the basis of assigned ST metaprogram. (C) Relative frequency of each metaprogram per sample, shown for ST-RELA, ST-Midline, and ST-YAP1 tumors. (D) Average relative activity of ST-EPN subpopulation-specific TF regulons by SCENIC. See also Figures S4 and S5; Table S2. Metaprogram Genes and Assignments, Related to Figures 2 and 4, Table S3. PF-EPN and ST-EPN Signatures, Related to Figures 2 and 4, Table S4. GO Term Enrichment Using Cell Population-Specific Signature and Top 30 Metaprogram Genes, Related to Figures 2 and 4, Table S5. Relative TF Activity and Metaprogram-Specific TFs, Related to Figures 2 and 4.

We analyzed eight ST-EPN samples, covering ST-RELA (n = 5), ST-YAP1 (n = 1), and two subsequent recurrent surgical samples of a patient with ST-EPN (BT1030, CPDM0785) classified as PF-A by methylation profiling despite clear supratentorial midline location of the primary tumor (see Fukuoka et al., 2018) (Figure 1A). Thus, we decided to refer to these samples as ST midline. Overall, we detected ten metaprograms across all patients defined by NMF and graph-based clustering (Figures 4A–4C, S4A, and S4D; Table S2. Metaprogram Genes and Assignments, Related to Figures 2 and 4, Table S3. PF-EPN and ST-EPN Signatures, Related to Figures 2 and 4, Table S4. GO Term Enrichment Using Cell Population-Specific Signature and Top 30 Metaprogram Genes, Related to Figures 2 and 4). Two metaprograms were again strongly associated with cell-cycle genes and were consequently termed ST-S-Phase (e.g., *KIAA0101*, *MCM2-7*, *PCNA*) and ST-G2M-Phase (*NUSAP1*, *CDC20*, *CDK1*) (Table S4). Those cycling cells occurred almost exclusively in ST-RELA tumors (Figure S4E). Importantly, a more differentiated metaprogram, ST-Ependymal-like, was characterized by ciliogenesis markers (e.g., *SPAG6*, *LRRC48*, *DNAAF1*), closely resembling mature ependymal cells in reference datasets and exhibiting strong transcriptional overlap with PF-Ependymal-like cells (Table S4). Interestingly, the matched ST-Midline pair exhibited a decrease of this differentiated subpopulation in the later (CPDM0785) as compared with the earlier (BT1030) recurrence (Figure 4C, p = 4.4 × 10^−28^, Fisher's exact test). ST-RELA, but not ST-YAP1 tumor cells additionally expressed two metaprograms that resembled undifferentiated progenitor cells. One of the undifferentiated programs present in ST-RELA strongly mapped to radial glial cells in both human and murine reference atlases and was thus termed ST-Radial-Glia-like (Figure S4F). This metaprogram exhibited only limited overlap with PF-NSC-like cells found in PF-EPNs (Table S2. Metaprogram Genes and Assignments, Related to Figures 2 and 4, Table S3. PF-EPN and ST-EPN Signatures, Related to Figures 2 and 4, Table S4. GO Term Enrichment Using Cell Population-Specific Signature and Top 30 Metaprogram Genes, Related to Figures 2 and 4). A second undifferentiated program in ST-EPN, ST-Neuronal-Precursor-like, was characterized by genes implicated in early neuronal fate determination (*STMN2/4*, *ELAVL4*, *NEUROD1*). Two more programs were shared among ST-RELA tumors but not in ST-YAP1 tumors, and were characterized by genes implicated in interferon signaling (ST-Interferon-Response, e.g., *ISG15*, *IFI6/27/30*, *IFIT1/3*) or strongly associated with glycolysis as well as hypoxia (ST-Metabolic, e.g., *LDHA*, *ENO2*, *PGK1*) (Table S2. Metaprogram Genes and Assignments, Related to Figures 2 and 4, Table S3. PF-EPN and ST-EPN Signatures, Related to Figures 2 and 4, Table S4. GO Term Enrichment Using Cell Population-Specific Signature and Top 30 Metaprogram Genes, Related to Figures 2 and 4). Another program was exclusively found in one ST-RELA tumor, MUV043, and was associated with connective tissue development and extracellular matrix organization (e.g., *DLK1*, *PCP4*, *ACPT*) (ST-RELA-variable, Figure 4C). ST-midline and ST-YAP1 tumors equally expressed specific and mutually exclusive metaprograms, which were termed ST-Midline and ST-YAP1, respectively. Analogous to PF tumors, additional snRNA-seq of matched patient tissue by 10X Genomics partially recapitulated expression signatures identified in fresh patient tissue by scSmart-seq2 (Figure S4G). Moreover, metaprograms derived from ST-RELA PDX and neurospheroid cell models more closely resembled transcriptional programs found in patients compared with adherent ST-RELA cell cultures (Figure S4H).

To identify potential downstream targets of *C11orf95-RELA* fusion gene products, we first scored a combination of “wild-type RelA” and “C11orf95-RelA fusion” target genes (Parker et al., 2014; Ozawa et al., 2018) in all supratentorial EPN subpopulations. These genes were expressed across all ST-RELA subpopulations and only showed moderate enrichment in ST-Metabolic, ST-Interferon-Response, and ST-RELA-Variable programs (Figure S5A). Scoring genes exclusively activated by the C11orf95-RELA fusion, but not wild-type RELA (Parker et al., 2014), showed a similar expression pattern (Figure S5B).

As a second, independent approach, we performed SCENIC analysis—informed by a more extensive RelA target gene list—to test TF activities across subpopulations. This indicated high RELA TF activity in all seven programs expressed in ST-RELA tumors (Figure S5C), whereas in YAP1- and the ST-Midline-specific programs the activation of RELA signaling was expectedly low. In addition, SCENIC analysis suggested distinct TF signatures for ST-Ependymal-like, ST-Midline, ST-YAP1, ST-RELA-Variable, and ST-Neuronal-Precursor-like subpopulations (Figure 4D and Table S5), the latter one sharing *NEUROG1* with PF-Neuronal-Precursor-like cells. In contrast, ST-Ependymal-like cells were characterized by a TF network (*RFX8*, *SRY*, *CAT*, *PAX8*, *GATA1*) that differed from that encountered in the PF-Ependymal-like counterpart (Figure 2E), potentially reflecting regional specificity of TF activity. Together, these data suggest an overarching role of RELA target gene expression across all subpopulations in ST-RELA but indicate that transcriptional diversification resulting in multiple cellular states is independent of C11orf95-RELA activity.

Taken together, scRNA-seq of ST-EPN demonstrated that ST-RELA tumors are characterized by highly proliferating, undifferentiated radial glia-like or neuronal-precursor-like cell types with only limited differentiation programs compared with PF-EPN (Figures 4C and S5D). ST-Midline and ST-YAP1 EPN groups exhibited even lower transcriptional complexity and harbored considerably smaller fractions of undifferentiated and proliferating cells, likely reflecting the milder disease course of these molecular ST-EPN groups.

### ST-EPN Metaprograms Predict Patient Survival and Can Be Therapeutically Targeted

We again applied RNA velocity to estimate a putative differentiation trajectory within ST-RELA tumors, which exhibited the highest intra- and intertumor heterogeneity among ST-EPN groups. As described above (Figures 4C and S5D), most cells within ST-RELA tumors reflect poorly differentiated progenitor-like cells, including radial glia-like and neuronal-precursor-like cells, while only a minority of cells expressed differentiated programs (e.g., ST-Ependymal-like). Consequently, RNA velocity did not support a clear developmental hierarchy within the undifferentiated populations (ST-Radial-Glia-like, ST-Neuronal-Precursor-like) (data not shown).

**Figure 5:**
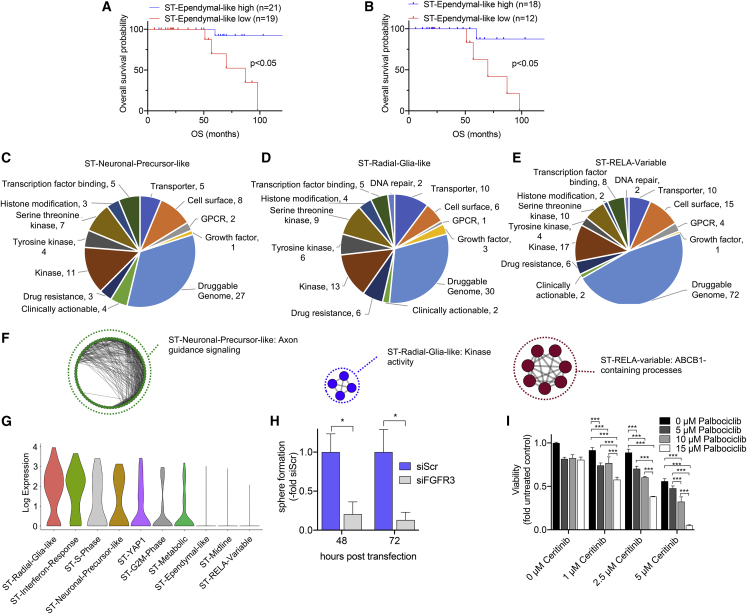
Survival Implications and Potential Vulnerabilities of ST-EPN Subpopulations (A and B) Overall survival (OS) stratification of ST (A) and ST-RELA (B) EPN tumors according to high or low relative expressions of top 30 genes for the ST-Ependymal-like metaprogram in bulk RNA expression data. Significance levels were determined by log-rank test. (C–E) Pie charts of candidate genes for therapeutic targeting, analyzed by integrating significantly differentially expressed genes of ST-Neuronal-Precursor-like (C), ST-Radial-Glia-like (D), and ST-RELA-variable (E) metaprograms with DGIdb. Gene hits in the “Druggable genome” and “Clinically actionable” references are shown. (F) Network maps of significantly enriched GO terms (p = 0.01, g:SCS Threshold) derived from G-Profiler pathway enrichment analysis of DGIdb gene hits in ST-Neuronal-Precursor-like, ST-Radial-Glia-like, and ST-RELA-variable metaprograms. (G) Log-transformed expression of *FGFR3* across ST-EPN metaprograms. (H) Relative sphere area at 48 h and 72 h post transfection of the ST-EPN cell model VBT242 with siFGFR3 or non-targeting siRNA (siScr). Sphere formation upon siFGFR3 KD is depicted relative to respective siScr controls. ^∗^p < 0.05, two-tailed Student's t test. Data are presented as mean ± SEM of triplicate values. (I) Viability of VBT242 cells upon 72-h combinatorial treatment with indicated concentrations ceritinib and palbociclib was determined by CellTiter-Glo assay. ^∗∗∗^p < 0.001, two-way ANOVA, Tukey's multiple comparisons test. Data are presented as mean ± SD of triplicate values. See also Figure S6 and Table S7.

We next investigated the implications of ST-EPN metaprograms with respect to risk stratification by clustering a large bulk RNA-seq patient cohort (n = 30) into high- and low-expressing groups. Even though the differentiated ST-Ependymal-like signature was only present in a minority of our ST-EPN scRNA-seq cohort, we found that several tumors in the bulk expression dataset scored highly for this transcriptional program. Analogous to PF-EPN, segregation according to high versus low ST-Ependymal-like signatures demonstrated that tumors highly expressing this program exhibited a significantly favorable prognosis compared with low-expressing tumors (Figures 5A and S6A). We also confirmed this in ST-RELA tumors separately (Figures 5B and S6B).

To identify potentially druggable vulnerabilities in ST-RELA, we next interrogated DGIdb with signature-specific genes and found *CCND2*, *HDAC2*, and *EFNA5* (ST-Neuronal-Precursor-like, Figure 5C and Table S7), *FGFR3*, *IGF2*, and *WNT7B* (ST-Radial-Glia-like, Figure 5D), and *RPS6KB2* and *EGFL7* as potentially druggable targets (ST-RELA-variable, Figure 5E) (Hong et al., 2018). Mapping of DGIdb hits in functional networks demonstrated that ST-Radial-Glia-like cells were associated with kinase activity-related processes, whereas ST-Neuronal-Precursor-like genes were strongly implicated in signaling circuitries regulating axon guidance mechanisms (Figure 5F and Table S7). The ST-RELA-variable program was characterized by mechanisms involving the major multidrug efflux transporter gene *ABCB1* (Figure 5F and Table S7).

As first-proof-of-principle functional validation experiments, we found that transient, siRNA-mediated KD of the ST-Radial-Glia-like program marker *FGFR3* (Figure 5G) led to significantly impaired sphere-formation capacity of *FGFR3*-expressing VBT242 cells (Figures 5H and S6C). Treatment with the FGFR inhibitor dovitinib effectively inhibited cell viability in VBT242 cells (Figure S6D). Likewise, ceritinib, a clinically approved ALK inhibitor that also inhibits the IGF2/IGF1R axis, another marker for ST-Radial-Glia-like cells (Figure S6E), was effective in reducing cell viability in this model (Figure S6F). Furthermore, simultaneous targeting of the ST-Neuronal-Precursor-like and ST-Radial-Glia-like programs (Figure S6E) with palbociclib (targeting the CDK4/6-CCND2 module) and ceritinib (targeting the IGF2/IGF1R axis), respectively, resulted in significantly reduced viability of VBT242 cells (Figure 5I) despite the fact that palbociclib as a single agent exerts predominantly cytostatic effects (Figure S6G).

Collectively, our data imply that the limited transition of undifferentiated ST-RELA radial-glia-like tumor cells into more differentiated, proliferatively inactive cell types might underlie the aggressiveness of this EPN group. Moreover, certain delineated transcriptomic signatures are of prognostic value and even define molecular vulnerabilities which appear feasible for clinical translation after first preclinical tests.

### Myxopapillary EPN Is Composed of Ependymal-like Cells and Undifferentiated Cells Resembling the PF-NSC-like Subpopulation

**Figure 6:**
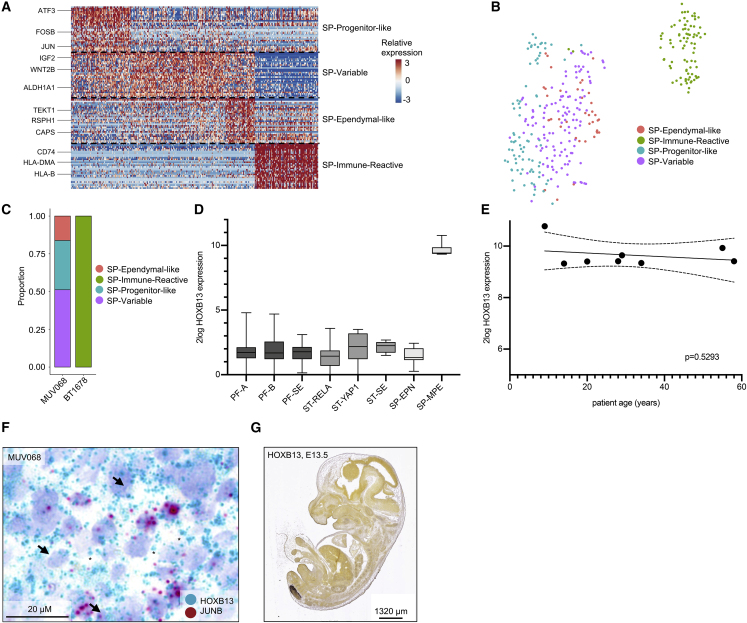
Intratumoral Heterogeneity in SP-MPE (A) Relative expression (color bar) across 333 malignant cells (columns) of the top 30 genes (row) for each SP metaprogram. (B) tSNE plot of all MPE tumor cells, colored on the basis of assigned metaprogram. (C) Relative frequency of each metaprogram per MPE sample. (D) Log expression of *HOXB13* RNA across all molecular EPN groups in bulk tumor cohort. (E) Correlation of relative *HOXB13* expression with SP-MPE patient age. Best-fit regression lines and 90% confidence bands are shown. Significance level was determined by linear regression. (F) RNA *in situ* hybridization of pan-MPE (*HOXB13*) and SP-Progenitor-like (*JUNB*) gene markers in FFPE tissue matched to scRNA-seq samples. Arrows and asterisks indicate representative cells positive for *HOXB13*, and *JUNB*, respectively. (G) Expression of *Hoxb13* in the developing mouse embryo (embryonic day 13.5 [E13.5]). *In situ* hybridization data were obtained from the Allen Developing Mouse Brain Atlas (Copyright Allen Institute for Brain Science, http://developingmouse.brain-map.org). See also Figure S7.

Following analysis of intracranial EPN, we analyzed spinal myxopapillary EPN (SP-MPE) samples by scRNA-seq and detected four shared subpopulations of tumor cells (Figures 1A, 6A, 6B, S7A, and S7B; Table S2). SP-Ependymal-like cells mapped to differentiated ependymal cell types in reference datasets and closely resembled the PF-Ependymal-like as well as ST-Ependymal-like programs. Interestingly, the undifferentiated SP-Progenitor-like population shared many marker genes with PF-NSC-like cells, suggesting a similar cell of origin for these tumors. A third metaprogram, termed SP-Immune-Reactive, was characterized by genes implicated in immunological processes (e.g., *HLA-DRA*/*DPA1*/*DRB1*/*DMA*, *CD74*, *CD14*, *B2M*) and did not confidently map to any developmental or adult brain cell type. While most cells of SP-MPE tumor BT1678 expressed this SP-Immune-Reactive program, SP-MPE tumor MUV068 exhibited higher metaprogram diversity and expressed the undifferentiated SP-Progenitor-like as well as the rather differentiated SP-Ependymal-like signatures and a third signature that was not found in BT1678, termed SP-variable (Figures 6C and S7A).

We furthermore identified high expression of the previously described transcription factor *HOXB13* (Barton et al., 2010) to be exclusive for SP-MPE (Figure 6D) and independent of age (Figure 6E). We next performed RNA-ISH in SP-MPE tissue sections of MUV068 and confirmed expression of this marker in all cancer cells, while only a small subpopulation of cells also stained positive for the SP-Progenitor-like marker *JUNB* in an interspersed manner (Figure 6F). Of note, spatial analysis of *HOXB13* expression in the developing mouse embryo showed that expression is limited to the most caudal parts of the spinal cord, the predominant locations for SP-MPE (Figure 6G).

We conclude that SP-MPE aberrantly recapitulates developmental processes spatially restricted to the caudal spinal region, and that patients share common transcriptional programs irrespective of patient age.

### Comparison of Metaprograms Reveals EPN-Specific as well as Pan-Glioma Shared Signatures

**Figure 7:**
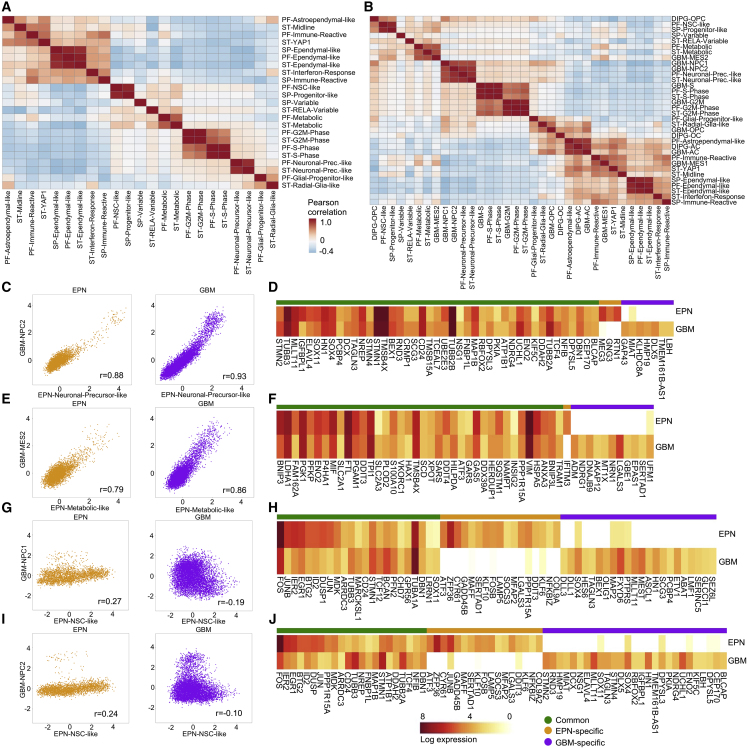
Intercorrelation of Metaprograms within EPN and across Other Glioma Types (A) Pairwise correlation of expression score of EPN metaprograms defined in each compartment and applied across cells from all compartments. (B) Pairwise correlation of expression score of metaprograms defined in EPN, DIPG, and GBM, and applied to cells from EPN. (C) Malignant cells (dots) from EPN and GBM scored for the EPN-Neuronal-Precursor-like (x axis) versus GBM-NPC2 (y axis) programs. Correlation coefficients, given as r values, are shown in the bottom right quadrants. (D) Aggregated log_2_-transformed gene expression in EPN-Neuronal-Precursor-like/GBM-NPC2 cells from each tumor class (rows) for metaprogram genes from either tumor (columns), with genes ordered into those common to EPN and GBM, or specific to either tumor type. (E) Analysis as in (C) for the EPN-Metabolic-like (x axis) versus GBM-MES2(y axis) programs. (F) Analysis as in (D) in EPN-Metabolic/GBM-MES2 cells. (G) Analysis as in (C) for the PF-NSC-like (x axis) versus GBM-NPC1 (y axis) programs. (H) Analysis as in (D) in PF-NSC-like/GBM-NPC1 cells. (I) Analysis as in (C) for the PF-NSC-like (x axis) versus GBM-NPC2 (y axis) programs. (J) Analysis as in (D) in PF-NSC-like/GBM-NPC2 cells. See also Figure S7.

We next assessed similarities and differences of transcriptional programs across all EPN groups. We scored tumor-group-specific metaprograms (n = 23) in all 4,401 malignant cells from our fresh Smart-seq2 dataset, irrespective of molecular group. Pairwise correlation of metaprogram expression scores revealed five clusters of metaprograms that were highly correlated and therefore similar across all EPN groups (Figure 7A): Cell-cycle-related programs (S and G_2_M phase) showed high similarity (average r = 0.98) between PF and ST samples. Likewise, the Ependymal-like programs identified in ST, PF, and SP tumors exhibited high pairwise correlation (average r = 0.92), indicating almost identical ependymal-like cell differentiation programs in all anatomical locations. Within undifferentiated cell populations, similar progenitor-like populations were identified in both PF- and SP-EPN (r = 0.92), but were different from stem-cell-like populations in ST-RELA or ST-YAP tumors. Importantly, the two neuronal-precursor-like populations found in PF-A and ST-RELA samples were highly correlated (r = 0.89), indicating related neuronal-like tumor cell trajectories in the two less favorable EPN groups. The metabolic populations in PF-A and ST-RELA were also highly correlated (r = 0.72), suggesting a shared subpopulation active in glycolysis and hypoxia-associated processes (*LDHA*, *PGK1*, *HK2*, *PGAM1*). In summary, our findings highlight that immature cancer cell types found in PF-EPN and ST-EPN exhibit very limited transcriptional overlap, pointing to a spatiotemporally-specific EPN stem cell niche despite their potential to give rise to very similar differentiated ependymal-like cancer cells.

As previous reports described a putative role of candidate EPN oncogenes in the generation of high-grade glioma-like tumors in murine models (Mohankumar et al., 2015; Johnson et al., 2010; Taylor et al., 2005), we next aimed at comparing developmental hierarchies of EPN with other high-grade gliomas using our primary, human tumor-derived scRNA-seq dataset. We first correlated all EPN metaprograms with other high-grade glioma metaprograms previously described in diffuse intrinsic pontine glioma (DIPG) and glioblastoma (GBM) (Filbin et al., 2018; Neftel et al., 2019) (Figures 7B, S7C, and S7D). Similarly, we scored these programs in our fresh Smart-seq2 dataset and analyzed pairwise correlation of metaprograms from different tumor types (Figure 7B). Interestingly, we found that the Neuronal-Precursor-like programs in EPN exhibited high similarity to the neural-precursor-like cell (NPC) programs described in GBM, suggesting similarly aberrant neuronal lineage gene expression in both of these tumor types (Figures 7C and 7D). In addition, the two metabolic programs in PF-EPN and ST-EPN strongly correlated with the Mes-2 program described in GBM (Figures 7E and 7F), indicating hypoxia-related response as a shared feature of both tumor types. On the contrary, the very immature PF-NSC-like signature found in PF-EPN showed very small overlap with neither NPC1 (Figures 7G and 7H) nor NPC2 (Figures 7I and 7J) programs in GBM, nor any program in DIPG, likely pointing toward different cells of origin. Furthermore, ependymal-like programs found in EPN did not resemble any mature program encountered in GBM, highlighting this program as a unique transcriptional EPN feature. In contrast, the intermediate PF-Astroependymal-like program found in EPN was highly correlated with the two astrocyte-like programs from both DIPG and GBM, indicating a common astrocytic lineage implicated in these programs.

Collectively, these data demonstrate partially shared transcriptional programs when comparing EPN signatures across all molecular groups as well as with other high-grade gliomas, but also indicate putatively different cells of origin and/or distinct spatiotemporal time points during human CNS development leading to these different classes of high-grade gliomas.

## Discussion

Our comprehensive single-cell analyses across all major molecular EPN groups, anatomical locations, and age groups demonstrate that EPNs are composed of cellular subpopulations that transcriptionally resemble normal brain cell development. We show that these tumor cell types are stalled in diverse differentiation states, and discover a trajectory originating from undifferentiated NSC-like or radial glia-like subpopulations toward three lineages of impaired neuronal-glial fate specification. Thus, our study supports the previously described role of aberrant radial glia-like cells as potential cells of origin for EPN (Johnson et al., 2010; Taylor et al., 2005) and reveals an additional layer of cell fates arising from these stem-like populations directly in human EPN samples. We describe three differentiation trajectories, including ependymal-like, glial-progenitor-like, and neuronal-precursor-like cells. Of these, the ependymal-like programs represent the most differentiated of all tumor cells and are predominantly found in prognostically favorable molecular groups. The transcriptomic program underlying this differentiation axis is characterized by TF networks (including *FOXJ1*) underlying a strong ciliogenesis signature, in keeping with previous work demonstrating high ciliogenic marker expression in ST-YAP1 and PF-B (Mack et al., 2018). In contrast, prognostically poor tumors harbor more undifferentiated cell populations.

Previous preclinical genome-wide analyses of human tumors and animal models had already pinpointed the importance of stemness features in the biology of EPN (Johnson et al., 2010; Ozawa et al., 2018; Taylor et al., 2005; Mack et al., 2018). We here describe the presence of an NSC-like cell at the root of PF-EPN and a radial-glia-like cell at the root of ST-RELA EPN. Our signatures partially overlap with programs identified in genetic mouse models of EPN (Mohankumar et al., 2015), thereby validating their findings in human tumors. Moreover, our analyses indicate a trend toward enrichment of undifferentiated programs and loss of more differentiated programs in recurrent tumors, potentially reflecting a selection process toward a more malignant transcriptomic profile upon tumor progression. It is also worth noting that we identify undifferentiated NSC-like programs in SP-MPEs, which—despite low tumor grade and a generally good prognosis—are well known for their potency of leptomeningeal metastasis (Rezai et al., 1996) in younger patients. This is surprising, but is in line with previously observed partially shared expression patterns between spinal, PF, and ST NSC, as well as NSC-derived EPN mouse models (Johnson et al., 2010; Taylor et al., 2005).

Similar to other stem-like tumor cell populations, neuronal-precursor-like cells are only identified in biologically aggressive EPN groups. In line with these findings, we have previously described induction of aberrant neuronal development processes by expression of the PF-A hallmark gene *CXorf67* (EZHIP) (Hubner et al., 2019). This neuronal-precursor-like program also exhibits a clear similarity to previously identified NPC programs in pediatric and adult glioblastoma (Neftel et al., 2019). This indicates that aggressive EPNs share transcriptionally similar cellular subpopulations with GBM, and validates previous reports that described candidate EPN oncogenes generating GBM-like tumors in mouse models (Mohankumar et al., 2015). However, despite the presence of similar subpopulations, the putative cell (or transcriptional state) of origin in EPN seems to be different from that in GBM.

Of note, our RNA velocity analysis suggests a unidirectional differentiation pattern within EPN, which parallels reports of similarly unidirectional hierarchies in adult GBM (Wang et al., 2019b; Fine, 2019). Further functional studies will need to determine whether EPN hierarchies truly represent unidirectional differentiation trajectories following aberrant NSC/radial glia-like development or still allow for some plastic interconversion between cellular states (Fine, 2019).

Our analysis also detects cell populations that validate prior findings describing metabolic processes linked to glycolysis and hypoxia, as well as immune regulatory mechanisms being prominent transcriptional programs in EPN (Preusser et al., 2005; Mack et al., 2015; Mohankumar et al., 2015). Whether these signatures reflect aberrant developmental processes stalled in EPN stem-like cells or rather represent dynamic metabolic/phenotypic changes in relation to the immune and non-immune tumor microenvironment will be investigated in future studies enriched for higher numbers of tumor-associated stromal cells.

Notably, analysis of an EPN bulk RNA-seq expression reference dataset reveals a profound effect of differentiation states on patient survival. We show that the observed biological and clinical differences in EPN may be caused by the presence of diverse differentiation states within tumors. Interestingly, our finding that the relative abundance of stem cell signatures predicts the clinical course of EPN rather than the abundance of cell-cycle activation also provides a possible explanation for the widely accepted fact that a higher tumor grade is not a reliable prognostic factor for intracranial EPN (Ellison et al., 2011).

We therefore also reasoned that improved therapeutic strategies for aggressive EPN groups should include inhibition of signaling circuitries maintaining these undifferentiated cell states. Our preliminary results of reducing stemness and cell viability by targeting more aggressive tumor cell subpopulations in EPN models show that this treatment strategy is promising, while recognizing that these efforts have to be expanded in the future.

Taken together, our study provides insights into the landscape of intratumoral heterogeneity and cellular hierarchies underlying EPN biology across all major molecular groups. Our data substantiate the radial glia/NSC EPN stem cell paradigm by providing whole transcriptome-scale insights into aberrant neurodevelopmental pathways driving EPNs in humans. Deconvolution of heterogeneous EPN subpopulations pinpoints key malignant transcriptomic signatures, which identify high-risk tumors and subsequently inform the development of more effective anti-EPN treatments.

## STAR★Methods

### Key Resources Table

**Table:** 

REAGENT or RESOURCE	SOURCE	IDENTIFIER
**Biological Samples**		

Human tumor tissue samples	This paper	N/A

**Chemicals**, **Peptides**, **and Recombinant Proteins**		

RPMI-1640 medium	Sigma	Cat# R6504
Fetal Calf Serum (FCS)	Biowest	Cat# S1400
Penicillin/Streptomycin	GEHealthcare	Cat# SV30010
Neurobasal Medium	GIBCO	Cat# 21103-049
N2/B27	GIBCO	Cat# 17502-048/17504-044
L-glutamine	Sigma	Cat# G8540
EGF	Sigma	Cat# E9644
FGF2	PeproTech	Cat# 100-18B
NeuroCult NS-A Basal Medium	STEMCELL Technologies	Cat# 05750
NeuroCult Proliferation Supplement	STEMCELL Technologies	Cat# 05701
bovine serum albumin (BSA)	Sigma	Cat# A8531
Neurobasalmedium A	Life Technologies	Cat# 10888-022
Heparin	Sigma	Cat# H0200000
Laminin	Sigma	Cat# L2020
Geltrex	Life Technologies	Cat# A1569601
Accutase	Stemcell Technologies	Cat# 07922
calcein AM	Life Technologies	Cat# C3100MP
TO-PRO3 iodide	Life Technologies	Cat# T3605
TCL buffer	Qiagen	Cat# 1031576
β-mercaptoethanol	Sigma	Cat# M6250
Vybrant DyeCycle Ruby Stain	Life Technology	Cat# V10309
RNAscope Hydrogen Peroxide	ACD	Cat# 322335
RNAscope 1X Target Retrieval Reagent	ACD	Cat# 322000
RNAscope Protease Plus	ACD	Cat# 322331
Gill’s Hematoxylin I	Statlab	Cat# HXGHE1LT
Vectamount Permanent Mounting Medium	Vector Laboratories	Cat# H-5000
Dimethyl sulfoxide (DMSO)	Sigma	Cat# 276855
Dovitinib	Selleck Chemicals	Cat# S1018
Ceritinib	Selleck Chemicals	Cat# S7083
Palbociclib	Selleck Chemicals	Cat# S1116
Wnt-C59	Selleck Chemicals	Cat# S7037
Panobinostat	Selleck Chemicals	Cat# S1030
KAPA HiFi HotStart ReadyMix (2X) 6.25ml	Kapa Biosystems	Cat# KK2602
Maxima H Minus Reverse Transcriptase	Life Technologies	Cat# EP0753
Recombinant RNAse Inhibitor (RRI)	Takara	Cat# 2313B
Betaine	Sigma	Cat# B0300
MgCl_2_	Sigma	Cat# M1028-10X1ML
Agencourt RNAClean XP beads	Beckman	Cat# A63987
Agencourt AMPure XP beads	Beckman	Cat# A63881

**Critical Commercial Assays**		

Mycoplasma Stain kit	Sigma	Cat# MP0025
Brain Tumor Dissociation Kit	Miltenyi Biotec	Cat# 130-095-942
Infinium Methylation EPIC BeadChip array	Illumina	Cat# WG-317-1001
RNAscope 2.5 HD Duplex Detection Kit	Advanced Cell Diagnostics (ACD)	Cat# 322430
Nextera XT DNA Library Preparation Kit (96 samples)	Illumina	Cat# FC-131-1096
NextSeq 500/550 High Output Kit v2.5 (75 Cycles)	Illumina	Cat# 20024906
10X Chromium Single Cell 3’ Reagent Kits v3	10X Genomics	Cat# PN-1000092
CellTiter-Glo® Luminescent Cell Viability Assay	Promega	Cat# G7573
Qubit dsDNA HS Assay Kit	Thermo Fisher Scientific	Cat# Q32854

**Deposited Data**		

Raw and analyzed data	This paper	GEO: GSE141460

**Experimental Models**: **Cell Lines**		

VBT211	This paper	N/A
VBT242	This paper	N/A
EP1NS	This paper	N/A
BT165	This paper	N/A
VBT96	This paper	N/A
BT214	This paper	N/A
EPD210FH	Brain Tumor Resource Lab	(www.btrl.org/)
BT214 PDX	(Brabetz et al., 2018)	N/A
BT165 PDX	(Brabetz et al., 2018)	N/A

**Oligonucleotides**		

Template-Switch Oligo (TSO)	Qiagen	Custom
Oligo dT primer	IDT	custom
ISPCR primer	IDT	custom
Accell Green Non-targeting siRNA	Dharmacon	Cat# D-001950-01-05
ON-TARGETplus SMARTpool siRNA, LGR5	Dharmacon	Cat# L-005577-00-0005
ON-TARGETplus SMARTpool siRNA, FGFR3	Dharmacon	Cat# L-003133-00-0005

**Software and Algorithms**		

DNA methylation-based classification of tumors of the central nervous system	Capper et al., 2018	www.molecularneuropathology.org, version 3.1.5
Conumee	N/A	http://bioconductor.org/packages/release/bioc/html/conumee.html
ImageJ	version 2.0.0-rc-69/1.52p,	RRID:SCR_003070
g:GOSt profiling online tool of G:Profiler	(Raudvere et al., 2019)	RRID:SCR_006809
EnrichmentMap plugin of Cytoscape	v3.7.2	RRID:SCR_003032
SPSS Statistics 25	IBM	
GraphPad Prism 8	GraphPad Prism Software	RRID:SCR_002798
ImageJ	v1.8.0	https://imagej.nih.gov/ij/
Bowtie	v0.12.7	http://bowtie-bio.sourceforge.net/bowtie2/index.shtml
Rsem	v1.2.19	(Li and Dewey, 2011)
R	v3.5.2	https://www.r-project.org
inferCNV	v.0.8.2	https://github.com/broadinstitute/infercnv/releases/tag/InferCNV-v0.8.2
Seurat	v2.3.4	https://satijalab.org/seurat/install.html
Pagoda2	v2.0.0.0.9003	(Fan et al., 2016)
NMF	v0.23.6	http://bioconductor.org/packages/release/bioc/html/conumee.html
clusterProfiler	v3.10.1	(Yu et al., 2012)
Harmony	v0.99.9	(Korsunsky et al., 2018)
velocyto.R	v0.6	(La Manno et al., 2018)
SCENIC	v.1.1.2-2	(Aibar et al., 2017)
cellranger	v3.0.2	https://github.com/10XGenomics/cellranger

**Other**		

RNAscope probe Hs-HOXB13	ACD	Cat# 400781
RNAscope probe Hs-JUNB	ACD	Cat# 534031-C2
RNAscope probe Hs-CD36	ACD	Cat# 536631-C2
RNAscope probe Hs-ATF3	ACD	Cat# 470861
96-well ultra-low attachment plates	Corning	Cat# COS3474

### Resource Availability

#### Lead Contact

Further information and requests for resources and reagents should be directed to and will be fulfilled by the Lead Contact, Mariella G. Filbin (mariella.filbin@childrens.harvard.edu).

#### Materials Availability

This study did not generate new unique reagents.

#### Data and Code Availability

The accession number for the data reported in this paper is GEO: GSE141460.

### Experimental Model and Subject Details

#### Human Subjects and Ethical Considerations

Patients with EPN and/or their legal representatives treated at Boston Children’s Hospital and at the Medical University of Vienna gave preoperative informed consent to participate in the study in all cases. The study was approved by the local Institutional Review Board (IRB) DFCI 10-417 (Boston Children’s Hospital and Dana-Farber Cancer Institute) and EK Nr. 1244/2016 (Medical University of Vienna). Clinical characteristics are summarized in Table S1.

#### Primary Cell Cultures

Patient-derived primary cultures used in this study included the ST- RELA models VBT211 (corresponding to MUV43), VBT242 (corresponding to MUV56), EP1NS, and BT165, as well as the PF-A models VBT96 (corresponding to MUV51), EPD210FH, and BT214. All cell models except for EPD210FH were established from fresh patient tumor specimen. EPD210FH cells were obtained from Brain Tumor Resource Lab (www.btrl.org/). For patient characteristics of primary tumors for derivation of cell models, please see Table S1. All cells were checked for Mycoplasma contamination on a regular basis in-house (Mycoplasma Stain kit, Sigma) and by a third-party service provider (GATC Biotech). Adherent cultures (VBT211-adh, VBT242-adh, VBT96-adh) were grown in RPMI-1640 medium (Sigma, R6504) supplemented with 10% fetal calf serum (FCS, Biowest) and 1% Penicillin/Streptomycin (GEHealthcare, SV30010) in tissue culture-treated flasks. Spheroid cultures (VBT211-sph, VBT242-sph) were grown in ultra-low attachment flasks (Corning, COR3815) in Neurobasal Medium (GIBCO, 21103-049) supplemented with 1X N2/B27 (GIBCO, 17502-048/17504-044), 1% Penicillin/Streptomycin, 2μM L-glutamine (Sigma, G8540), 20 ng/ml EGF (Sigma, E9644), and 20 ng/ml FGF2 (PeproTech, 100-18B). EPD210FH and BT214 cells were grown in NeuroCult NS-A Basal Medium (STEMCELL Technologies) supplemented with NeuroCult Proliferation Supplement (STEMCELL Technologies), 2mM L-glutamine 1% Penicillin/Streptomycin, 75ng/ml bovine serum albumin (BSA) and 20ng/ml of EGF (Peprotech) and FGF-basic (Peprotech). EP1NS cells were grown in Neurobasalmedium A (Life Technologies) supplemented with 1μg/ml of Heparin (Sigma), 2mM L-Glutamine and 20ng/ml of EGF and FGF-basic. EPD210FH and EP1NS cells were grown in T25, T75 and T150 tissue culture flasks (TPP). Flasks were additionally coated with Laminin (L2020, Sigma) for EPD210FH cells. For EP1NS cells, flasks were coated with Geltrex (A1569601, Life Technologies). BT214 cells were grown as spheroids in ultra-low attachment cell culture flasks (CLS3815, Corning). All cell models were grown at 37°C with 5% CO_2_ and were authenticated by methylation array.

#### Patient-derived Xenografts (PDXs)

PDX models of BT214 and BT165 were generated and obtained from a previous study (Brabetz et al., 2018).

### Method Details

#### Tumor Tissue Collection and Dissociation

##### Live Cell Isolation from Fresh Tissue

Fresh tumor tissue was collected at the time of surgery at Boston Children’s Hospital and Medical University of Vienna and processed immediately. Tumor tissue was dissociated mechanically followed by papain-based enzymatic digestion for 30 min at 37°C using a Brain Tumor Dissociation Kit (Miltenyi Biotec). Single-cell suspensions were filtered through a 70 μm strainer, centrifuged at 500 g for 5 min, and re-suspended in phosphate-buffered saline (PBS) supplemented with 1% bovine serum albumin (BSA, Sigma, A8531).

##### Nuclei Isolation from Frozen Tissue

Nuclei from fresh, snap-frozen tumor tissue as well as from frozen PDX cell pellets (Brabetz et al., 2018) were isolated as previously described (Slyper et al., 2019). Tissue was thawed briefly and immediately lysed on ice for 5 min under constant mechanical dissociation using surgical scissors. Single-nuclei suspensions were filtered using a 40 μm strainer, centrifuged at 500 g for 5min, and re-suspended in PBS supplemented with 1% BSA (Smart-seq2), or 0.05% BSA (10X Genomics).

##### Cell Models

EPN-derived spheroid and adherent cell models were dissociated to single-cell suspensions at the Medical University of Vienna and DKFZ by accutase-based (Stemcell Technologies, 07922) enzymatic digestion.

#### FACS-Sorting

##### Live Cells

Single-cell suspensions derived from fresh tumor tissue as well as EPN-derived cell models were re-suspended in PBS + 1% BSA. Tumor tissue-derived cell suspensions were stained with 0.2 μM calcein AM (Life Technologies, C3100MP) and 0.5 μM TO-PRO3 iodide (Life Technologies, T3605) for 10 min at room temperature in PBS + 1% BSA. Single-cell sorting was performed on a SH700 sorter (Sony) using 488 nm (calcein AM, 530/30 emission filter) and 633 nm (TO-PRO3, 665/30 emission filter) lasers. Unstained and single-stained controls were included for all tumors. Viable tumor cells were selected by positive staining for calcein AM as well as negative staining for TO-PRO3. Doublet discrimination was performed by stringent singlet-gating in the back scatter area (BSC-A) versus back scatter width (BSC-W) setting. Singlet, viable tumor cells were sorted into 96-well plates (company, number) containing pre-chilled TCL buffer (Qiagen, 1031576), immediately snap frozen on dry ice and stored at -80°C until whole transcriptome amplification, library preparation and sequencing.

##### Nuclei

Single-nuclei suspensions derived from frozen tumor tissue were re-suspended in PBS + 1% BSA and stained with 0.5 μM Vybrant DyeCycle Ruby Stain (Life Technology, V10309) immediately before FACS sorting. Unstained controls were included for all samples. Single-nucleus sorting was performed on a SH700 sorter using the 633 nm laser (Ruby Stain, 665/30 nm emission filter). Intact nuclei were selected by positive staining for Ruby Stain. Doublet discrimination was performed by stringent singlet-gating in the Ruby Stain area versus Ruby Stain width setting. Singlet nuclei were sorted into 96-well plates containing pre-chilled TCL buffer and 1% β-mercaptoethanol, immediately snap frozen on dry ice and stored at -80°C until processed for whole transcriptome amplification, library preparation and sequencing.

#### scRNA-seq and snRNA-seq Data Generation

##### Smart-seq2

Whole transcriptome amplification, library preparation, and sequencing of single cells and single nuclei was performed following the Smart-seq2 modified protocol as previously described (Venteicher et al., 2017; Hovestadt et al., 2019; Neftel et al., 2019; Filbin et al., 2018; Slyper et al., 2019).

After single-cell sorting, RNA was purified with Agencourt RNAClean XP beads (Beckman). Oligo-dT primed reverse transcription was performed using Maxima H Minus reverse transcriptase (Life Technologies) and locked TSO oligonucleotide (Qiagen). This was followed by 20 cycle PCR amplification using KAPA HiFi HotStart ReadyMix (KAPA Biosystems) and subsequent Agencourt AMPure XP bead (Beckman) purification. Library tagmentation was performed using the Nextera XT Library Prep kit (Illumina). Libraries from 768 cells with unique barcodes were combined and sequenced on a NextSeq 500 sequencer (Illumina) using a NextSeq 500/550 High Output Kit v2.5 (Illumina).

##### 10X Genomics

Single nuclei were processed using the microfluidics-based 10X Chromium Single Cell 3’ Reagent Kits v3 (10X Genomics, PN-1000092). Briefly, 10,000 nuclei were added to each chip channel and partitioned into Gel Beads-in-emulsion (GEMs) on the Chromium Controller, followed by nuclei lysis and barcoded RNA reverse transcription. Library preparation was performed after breaking of single-nuclei emulsions, and included cDNA amplification, fragmentation, ligation of sample index, as well as addition of Illumina P5/P7 adapters.

#### DNA Methylation Profiling

All patient tumor samples that were processed for single-cell and single-nuclei sequencing, were also characterized by Infinium Methylation EPIC BeadChip array (Illumina) according to the manufacturer’s instructions. Methylation data for each patient sample were generated from corresponding formalin-fixed, paraffin-embedded tissue. EPN groups were predicted using a web-platform for DNA methylation-based classification of tumors of the central nervous system (www.molecularneuropathology.org, version 3.1.5) (Capper et al., 2018). Resulting assignment of samples to SP-MPE, ST-RELA, ST-YAP1, PF-A, PF-B EPN groups, as well as to further PF-A and PF-B groups were used as reference for all downstream analyses. CNA analysis from methylation profiling data was performed using the conumee Bioconductor package and compared to those predicted from the single-cell data.

#### RNA In Situ Hybridization

FFPE tissue sections (4 μm) from SP-MPE (MUV68) and PF-A (MUV013, MUV021, MUV038) tumors were obtained from the Institute of Neurology, Medical University of Vienna. Sections were mounted on Superfrost Plus glass slides (Fisher Scientific, 12-550-15) and stored at -80°C. Sections were stained using RNAscope 2.5 HD Duplex Detection Kit (Advanced Cell Diagnostics (ACD), 322430) according to the manufacturer’s instructions and as described previously (Neftel et al., 2019). Sides were baked at 60°C for 1 h and deparaffinized with xylene and ethanol. Dehydrated tissue was pretreated with RNAscope Hydrogen Peroxide (ACD, 322335) for 10 min at room temperature. Target retrieval was performed with RNAscope 1X Target Retrieval Reagent (ACD, 322000) in a steamer (Braun, FS20) at 99°C for 15 min, followed by treatment with RNAscope Protease Plus (ACD, 322331) at 40°C for 30 min. Hybridization probe combinations were prepared by diluting C2 probe (red, alkaline phosphatase) 1:50 into C1 probe (green, horseradish peroxidase). ACD RNAscope probes used included Hs-HOXB13 (ACD, 400781), Hs-JUNB (ACD, 534031-C2), Hs-CD36 (ACD, 536631-C2), and Hs-ATF3 (ACD, 470861). Probes were added to the tissue sections and hybridized for 2 h at 40°C. Ten amplification steps were performed following the instructions provided in the RNAscope 2.5 HD Duplex Detection Kit protocol. Red signal and green signal were detected after amplification steps 6 and 10, respectively. Tissue was counterstained with Gill’s Hematoxylin I (Statlab, HXGHE1LT) for 30 s at room temperature followed by bluing with 0.02% ammonia water and mounting with Vectamount Permanent Mounting Medium (Vector Laboratories, H-5000). Microscopic images of stained tissue sections were taken on a DMi8 brightfield microscope (Leica Microsystems) using a 40X magnification lens and LAS-X software (Leica Microsystems). Images were processed using ImageJ (version 2.0.0-rc-69/1.52p, RRID:SCR_003070).

#### siRNA Knockdown (KD) and Sphere Formation

VBT96 and VBT242 cells were transfected with siRNA targeting LGR5 (ON-TARGETplus SMARTpool siRNA, L-005577-00-0005, Dharmacon, Lafayette, CO, USA) or FGFR3 (ON-TARGETplus SMARTpool siRNA, L-003133-00-0005, Dharmacon, Lafayette, CO, USA) or non-targeting siRNA (Accell Green Non-targeting siRNA, D-001950-01-05, Dharmacon, Lafayette, CO, USA) according to manufacturer’s instructions. Transfection rates were controlled via autoflourescence of the siRNA constructs and rates over 99% were considered sufficient. Following incubation for 4 h, cells were washed and plated in technical quadruplicates at a density of 10^3^/well in 96-well ultra-low attachment plates (Corning, COS3474, NY, USA) in Neurobasal Medium (GIBCO, 21103-049) supplemented with 1X N2/B27 (GIBCO, 17502-048/17504-044), 2μM L-glutamine (Sigma, G8540), 20 ng/ml EGF (Sigma, E9644), and 20 ng/ml FGF2 (PeproTech, 100-18B). Cells were then monitored by live cell imaging with a Visitron Systems live cell microscope (Puchheim, Germany) for 72 h at 37°C with 5% CO_2_. At indicated timepoints the size of neurospheres (area in 2D) was measured utilizing ImageJ-software (Ver 1.8.0) and compared to respective non-targeting controls. Differences in sphere formation between non-targeting control and targeting siRNA at indicated timepoints were tested for statistical significance by student’s t-test. The depicted graphs show a representative experiment of two biological replicates, performed in technical triplicates. Values are represented as mean ± standard error of the mean (SEM). For statistical analysis, two-tailed student’s t-test was performed. Significance values are given as asterisks and signify ^∗^ p<0.05, or ^∗∗∗^ p<0.001. Groups compared are indicated as horizontal brackets.

#### Drug Sensitivity Experiments

In order to test sensitivity pf EPN ell models to inhibitors that target identified vulnerabilities, VBT96 and VBT242 were seeded at a density of 4x10^3^ cells/well in 96 well plates in the respective standard culture conditions indicated previously. After 24 h recovery time, cells were treated in triplicates with indicated drug concentrations for 72 h. Cell viability was tested by CellTiter-Glo® Luminescent Cell Viability Assay (Promega, G7573, Madison, WI, USA) according to manufacturer’s instructions and luminescence signals were measured with a Tecan infinite 200Pro plate reader. Dose-response curves and IC50 values were determined using Graph Pad Prism software (Version 8) based on a non-linear regression model of log (inhibitor) versus response for variable slope with four parameters. Sphere forming capacity was tested in VBT96 upon panobinostat incubation in in 24-well ultra-low attachment plates (Corning, COS3474, NY, USA) in Neurobasal Medium (GIBCO, 21103-049) supplemented with 1X N2/B27 (GIBCO, 17502-048/17504-044), 2μM L-glutamine (Sigma, G8540), 20 ng/ml EGF (Sigma, E9644), and 20 ng/ml FGF2 (PeproTech, 100-18B). Cells were seeded at a density of 2x10^4^ per well in duplicates and incubated with 50nM panobinostat after seeding. Sphere formation was evaluated by light microscopy (NIKON 7200 and Nikon 105mm F2.8 Macro lens) after 96 h and sphere size was quantified utilizing ImageJ-software (Ver 1.8.0) and compared to respective controls. Statistical differences between single and combinational treatments were determined by student’s t-test. The depicted graphs show a representative experiment of two biological replicates, performed in technical triplicates. For statistical analysis of combinatorial drug treatments, 2way ANOVA, Tukey’s multiple comparisons test was performed. Values are represented as mean ± standard deviation (SD). Significance values are given as asterisks and signify ^∗∗∗^ p<0.001. Treatment conditions compared are indicated as horizontal brackets.

#### scRNA-seq Data Processing

scRNA-seq data was pre-processed as previously described with some modifications (Neftel et al., 2019). Raw sequencing reads were aligned to hg19 genome by bowtie, and gene counts were quantified using RSEM as transcript-per-million, or TPM (Li and Dewey, 2011). Expression levels were calculated as *Ei*,*j* = log2(TPM*i*,*j*/10 + 1) for gene *i* in sample *j*. TPM values were divided by 10 for better approximation of the estimated complexity of single-cell libraries, which is in the order of 100,000 transcripts. To filter out low-quality cells, we removed cells with less than 2,000 detected genes or an average housekeeping expression level below 2.5. To filter out low-expressed genes, we removed genes with TPM greater than 10 in less than 10 cells. For the remaining cells and genes, we calculated the aggregate expression of each gene as *Ea*(*i*) = log2(average(TPM*i*,*1*…*n*) + 1). In each anatomical location (PF, ST, and SP), we defined relative expression as centered expression levels, *Eri*,*j* = *Ei*,*j* − average[*Ei*,*1*…*n*] for the remaining cells and genes. On average, we detected ∼4,600 genes per cell.

#### Identification of CNAs in Single-Cell Data

Copy number alterations (CNAs) were estimated as previously described by computing a moving average of the relative expression (Tirosh et al., 2016). Genes were sorted based on their chromosomal location and the mean relative expression of a sliding window of 100 genes within each chromosome were quantified. The presence of CNA was determined with hierarchical clustering of the single-cell copy-number profiles within each sample with 190 copy-number profiles determined from two non-malignant cell types (microglia/macrophages and tumor-associated oligodendrocytes) (Filbin et al., 2018). For the majority of tumors (16/20), most of the cells exhibited clear evidences of CNAs and did not cluster with the spike-in non-malignant cells.

#### Identification of Non-malignant Cell Types

T-distributed stochastic neighbor embedding (tSNE) embedding was generated using Seurat’s implementation of principal component analysis (PCA) and tSNE (Butler et al., 2018). Briefly, highly variable genes were selected based on overdispersion of genes in each gene group binned with aggregate expressions. Then relative expression values of these highly variable genes were used for PCA and top 18 principal components were selected for determining tSNE embeddings. Graph-based clustering implemented in Seurat was used to cluster cells and these clusters were well separated in the tSNE embedding. The majority of clusters contained cells from a single patient, but six clusters included cells from multiple patients. One of these clusters exhibited expression of cell-cycle related genes (*CDC20*, *CCND1*, and *TOP2A*), indicating enrichment with proliferating cells. The other five clusters showed high expression of marker genes for non-malignant cell types, including microglia (*CD14*, *FCER1G*, *CSF1R*), T cells (*CD3E*, *CD4*, *CD8A*), OPCs (*OLIG1*, *APOD*, *PDGFRA*), oligodendrocytes (*MBP*, *PLP1*, *MOG*), and endothelial cells (*IFITM1*, *CAV1*, *TM4SF1*). These cells were also classified not to possess CNAs.

#### Integrated Definition of Malignant Cells

We combined CNA classification with transcriptomic-based clustering and expression of non-malignant marker gene to identify malignant and non-malignant cells. Non-malignant cells showed high expression of specific marker genes (see gene list in the previous section) as well as no apparent CNAs. Malignant cells included those which were not part of the clusters with high expression of markers for non-malignant cell types and were classified to harbor CNAs. Cells with discordant classifications by marker gene expression and CNA detection were excluded from downstream analysis except for cells in MUV006 and MUV018. Most cells in MUV006 exhibited no apparent CNA, but were not part of non-malignant cell populations, so these cells were treated as malignant cells. Although cells in MUV018 showed no apparent CNAs, they closely clustered with cells from MUV014, which exhibited clear CNAs. In addition, cells in MUV018 were not part of non-malignant cell populations, so the majority of cells in MUV018 were also treated as malignant cells.

#### NMF Programs and Cellular Hierarchies

Non-negative matrix factorization (NMF) was utilized to derive transcriptional programs from relative expression data (with negative values converted to zero) as previously described (Hovestadt et al., 2019; Filbin et al., 2018). NMF programs were determined for malignant cells from each sample individually. The top 10,000 over-dispersed genes, as determined by PAGODA2 (Fan et al., 2016), were used in the NMF analysis. The number of factors were set to 10 for both PF and ST samples and 4 and 6 for BT1678 and MUV068. A relatively large number of factors were selected to avoid omitting major NMF factors. Since redundant NMF programs were merged into a single metaprogram, the final metaprogram was also not sensitive to the initially chosen number of factors. Top 30 genes with the highest NMF weights from each resulting NMF factor were selected to represent that factor. All single cells within each anatomical tumor compartment (PF, ST, and SP) were then scored for these NMF programs (top 30 genes). Within each compartment, NMF programs were clustered with hierarchical clustering (distance metric: one minus Pearson correlation; linkage: Ward’s linkage) on the scores for each program (Figures S1A and S4A). Only NMF programs clusters with an average correlation coefficient greater than 0.5 were retained, while individual NMF programs that did not cluster with any other program were manually inspected to keep those with strong association with known cell types or functional pathways. This revealed nine correlated sets of programs for all PF samples, ten for ST samples and four for SP samples. These correlated programs were then merged into metaprograms by selecting the top 30 genes with the highest average NMF weight within each correlated program set and resulted in a total of 23 compartment-specific metaprograms (Table S2).

#### Generation of Single-Cell Expression Scores

Single-cell expression scores were computed in a similar way as described previously (Hovestadt et al., 2019). Given a set of genes (*Gj*) for a NMF program or metaprogram, a score, SC*j*(*i*), which quantifies the relative expression of *Gj* for each cell *i*, was calculated as the average relative expression (Er) of the genes in *Gj*, compared to the average relative expression of a control gene set *G*cont: SC (*i*) = average[*Er*(*G*, *i*)]−average[*Er*(*G*cont, *i*)]. For each considered gene, the control gene set contains 100 genes with the most similar aggregate expression level to that gene. Therefore, the control gene set represents a comparable distribution of expression levels to that of the considered gene set, and the control gene set is 100-fold larger.

Single cells of each compartment were assigned to different cell subpopulations based on the maximum expression score for respective compartment-specific metaprograms. Cells were grouped into cycling and non-cycling based on maximal scores of S and G2M programs (larger and smaller than 1, respectively). For the pan-compartment analysis of all malignant EPN cells, expression values were re-centered across all compartments and expression scores were computed for each of the 23 metaprograms. The pairwise correlation of expression scores is shown in Figure 7A.

#### Cell Subpopulation-Specific Signature Genes

Signature genes were identified for each cell population that was assigned to a metaprogram using Wilcoxon rank sum test implemented in Seurat. Briefly, one cell subpopulation was compared with all other cell subpopulations and log transformed uncentered expression levels were used. For each cell subpopulation, genes that (1) showed Bonferroni-adjusted p value < 0.05, (2) showed at least 2-fold mean difference, and (3) were expressed by at least half the number of cells in this cell subpopulation were selected as signatures genes for that particular cell subpopulation (Table S3).

#### Characterization of Metaprograms

Besides manually inspecting underlying gene signatures of each metaprogram, we characterized the metaprograms by three complementary approaches as previously described (Neftel et al., 2019). (1) First, we tested for enrichment of described gene sets (Gene Ontology biological processes, molecular function, cellular component) in each metaprogram using a hypergeometric test (Table S4; Yu et al., 2012). (2) Second, we determined single-cell expression scores of non-malignant cell types for each of the metaprogram. We collected scRNA-seq data for non-malignant brain cells from multiple human and mouse brain atlas dataset (La Manno et al., 2016; Nowakowski et al., 2017; Zeisel et al., 2018). For each source, we aggregated cells by their reported cell type annotation, defined the median expression profile of each cell type (or used the respective data from the original study) and quantified expression score for cell types as described above. (3) Third, we characterized the expression similarities (Pearson correlations) between all non-malignant cell types from each brain atlas reference dataset used in (2) and the median profiles of all malignant cells mapping to each metaprogram. Only cell population specific genes (see earlier methods) were pooled and used for Pearson correlation calculation to minimize background correlation. (Figures S1E and S4F).

#### Graph-Based Clustering with Data Harmonization

We adapted graph-based clustering with data integration as an independent identification of cellular clusters and gene signatures. Highly variable genes were selected using Seurat (see earlier Methods). Then the relative expression values of these highly variable genes were used for PCA analysis. To separate sample-specific biological differences (i.e. unique combination of genetic and epigenetic alternations that are specific to each tumor) from cell type and state-specific biological variations, an efficient data harmonization method named Harmony was applied to the first 100 principle components with default parameters for data integration from multiple samples (Korsunsky et al., 2018; Korsunsky et al., 2019). Harmony learned and applied a linear adjustment function to generate a corrected embedding that is robust to sample-specific effects. The first 20 Harmony corrected dimensions were selected for quantifying tSNE embeddings. Cells were then grouped by expression of metaprograms (Figures 2B and 4B), and cells that were from different samples or compartments, but expressed similar metaprogram, were mixed together (Figures S1C and S4C). A second graph-based clustering approach implemented in Seurat was also utilized to group cells into subpopulations and the resulting clustering of cells looked remarkably similar to metaprogram-assigned clusters (Figures 2B, 4B, S1D, and S4D).

#### RNA-Velocity Analysis

The raw reads of each sample were first aligned to hg38 genome using hisat2 (Kim et al., 2015). Velocyto.py command line tool was applied to the resulting bam files to annotate spliced, unspliced and spanning reads based on gencode v27 gene annotation. Velocity was estimated using R implementation of a gene-relative model that combines KNN pooling with the gamma fit on the basis of extreme quantiles, with default parameters (La Manno et al., 2018). The resulting velocity estimate was projected onto the embedding of first two principle components for visualization (Figure 3A).

#### Lineage and NSC Score in PF Tumors

Cells were first ordered by their NSC-scores, defined as expression score of the PF-NSC-like program minus the maximal expression score of the two programs representing major differentiation trajectories (PF-Glial-Progenitor-like and PF-Ependymal-like program), and non-NSC-like cells were further classified by a lineage score distinguishing the Glial-Progenitor-like from Ependymal-like lineages (Figure S2B).

#### Gene Regulatory and TF Network Reconstruction

To characterize underlying gene regulatory network and transcription factor activities in our scRNA-seq dataset, the single-cell regulatory network inference and clustering (SCENIC) package was employed to identify gene regulatory modules and retain those with a cis-regulatory binding motif for upstream regulators (Aibar et al., 2017). Coexpression modules between TFs and putative target genes were estimated by GENIE3, followed by cis-regulatory motif analysis using RcisTarget and pruning of indirect targets lacking motif binding site. The resulting regulatory module (regulons, modules of target genes co-expressed with TFs and enriched with motifs for correct upstream regulators) activities in each cell were then binarized by AUCell. For each cell subpopulation, average relative activity of each TF regulon was also aggregated as proportions of cells with active regulon for that TF (Table S5). For each metaprogram, the most active TF regulons (average relative activity above 0.5) with average relative activity at least 50% greater than in any other metaprogram were selected as top specific TFs for each metaprogram (Figures 2E and 4D; Table S5).

#### Integration of the 10X Genomics Data

A second dataset from frozen tumors MUV006, MUV013, MUV014, MUV043, MUV051, and MUV056 was generated by the 10X-Genomics platform. Raw reads were aligned to hg38 genome and Unique Molecular Identifier (UMIs) were counted by cellranger using pre-mRNA gene annotation with intronic regions. Due to large variability in the number of cells collected from each sample, 3000 cells were randomly selected from each sample and their gene counts were pooled to construct a single count matrix (18,000 cells in total). Two criteria were applied to filter out low quality or cells: (1) number of detected genes and (2) proportion of UMIs mapped to mitochondrial genes. Cells with either total number of genes less than half or greater than twice the mean number of genes detected across cells or proportion of mitochondria UMIs greater than 5% were excluded from subsequent analysis. Relative expression values of highly variable genes were used for determining PCA and tSNE (see earlier Methods). Top 19 principal components were used for graph-based clustering, and expression of marker genes for normal cell types (see earlier Methods) were examined in each cluster. Two clusters of cells that express microglia/macrophage marker genes were then excluded from subsequent analysis. About 20% of cells were excluded during pre-processing.

#### Comparison of 10x and Smart-seq2 Results

Malignant cells from 10X were separated into PF and ST compartments and analyzed similarly. Relative expression values for highly variable genes were used for PCA, tSNE and graph-based clustering, with data integration by Harmony (see earlier Methods). Cells in each population were aggregated and log2 transformed. Median expression profiles were quantified for each cell population in the 10X dataset. Pairwise expression similarities (Pearson correlation) between each cell population in 10X and each cell population in Smart-seq2 (see earlier Methods) for pooled signature genes were determined in the PF and ST compartments. Each cell population in 10X was assigned to one or two metaprograms based on high correlation, indicating that expression signatures derived from 10X recapitulated metaprograms defined in the Smart-seq2 dataset.

#### Targetable Metaprogram-Specific Pathways

Significantly differentially expressed genes of identified EPN metaprograms were loaded into the g:GOSt profiling online tool of G:Profiler (Raudvere et al., 2019) (RRID:SCR_006809) and queried for functional enrichment of terms derived from GO (molecular function, cellular component, biological process), Kyoto Encyclopedia of Genes and Genomes (KEGG), and REACTOME databases. Furthermore, significantly overexpressed genes were used to query the Washington University Drug Gene Interaction database (DGIdb, v3.0.2, RRID:SCR_006608), focusing on expert-curated collections of druggable genes to identify metagene-specific candidate therapeutic targets (Cotto et al., 2018). DGIdb hits were again loaded into G:Profiler and queried for functional GO term enrichment. Multiple testing correction was applied using the SCS Threshold algorithm with a significance cut-off of p<0.01. Significantly enriched terms were imported into the EnrichmentMap plugin of Cytoscape (v3.7.2, RRID:SCR_003032) to generate functional network maps.

#### Pan-Glioma Metaprogram Comparison

To enable a direct comparison among EPN, DIPG, and GBM, we scored all cells in the EPN study (all EPN groups combined) and previous DIPG and GBM studies respectively for metaprograms identified from tumor types. Pairwise correlation of the resulting expression scores was shown in Figures 6B, S7C, and S7D. Like the pan-compartment comparison of EPN metaprograms, we utilized hierarchical clustering to group similar programs together, revealing commonality/specificity of expressions of metaprograms in EPN, DIPG, and GBM (Figures 6B, S7C, and S7D). We first uncovered metaprograms with astrocytic expression features in all three tumor types. We also identified the most similar expression programs expressed in both EPN and GBM, involving neuronal differentiation and glycolysis/hypoxia-reaction. We then demonstrated specificity of less differentiated metaprogram (PF-NSC-like in EPN and NPC1/2 in GBM) in either tumor, representing potentially distinct stem-like progenitors.

To further compare the expression programs between EPN and GBM, we defined tumor type-shared and -specific genes for the similar programs (Neuronal-Precursor-like/NPC2 and Metabolic-like/MES2) as well as progenitor-like programs (PF-NSC-like and NPC1/2) within each tumor. For top genes from pairs of these metaprograms, we aggregated their median expression in each cell population within each tumor and compared their expression between EPN and GBM. We then defined common genes as those with aggregated expression above 3 in both tumors, EPN-specific genes as those with aggregated expressions above 3 in EPN and differences in expression above 2 between EPN and GBM, GBM-specific genes as those with aggregated expression above 3 in GBM and differences in expressions above 2 between GBM and EPN. The resulting comparison of expression profiles of these genes were shown in Figures 6D, 6F, 6H, and 6J.

### Quantification and Statistical Analysis

#### Statistical Analysis

Statistical tests were performed using SPSS Statistics 25 (IBM), GraphPad Prism 8, or R. For computational as well as cell/molecular biological methods, statistical analyses are described in detail in respective methods chapters as well as indicated in figure legends and in corresponding results sections. Significance values are given in figures, figure legends, and/or the results section. Alternatively, significance levels are indicated as asterisks, signifying as ^∗^ p<0.05, ^∗∗∗^ p<0.001. Treatment conditions compared statistically are indicated by horizontal brackets in figures above respective data points. Cell viability curves for the different inhibitors and siRNA KD data were calculated from at least two independent experiments, performed as triplicates for each condition. Data points represent mean values ± SD or SEM, depending on the experiment performed and specified in figure legends and corresponding methods sections. In boxplots, boxes show the median and interquartile range, whiskers show minimum and maximum values. For G-Profiler pathway enrichment analysis of DGIdb gene hits, multiple testing correction was applied using the SCS Threshold algorithm with a significance cut-off of p<0.01.

#### Survival Analyses

Survival estimates were analyzed with SPSS Statistics 25 (IBM) and GraphPad Prism 8 (GraphPad Prism Software; RRID:SCR_002798). Univariate Models were calculated by the Kaplan-Meier Method and tested for significance by log-rank test. For multivariate testing we used multivariable Cox models. We next established the effect of the biomarkers (Ependymal, Neuronal, PFA-PFB-PF-SE) and clinical variables (Radiation therapy, Resection type) using multivariable Cox models and a two-step approach. First, we estimated the hazard ratios for a complete multivariable Cox model (including PFA-PFB-PF-SE, Table S1), and then re-estimated the hazard ratios with a multivariable Cox model excluding PFA-PFB-PF-SE (Table S5).

We did not observe relevant changes in the hazard ratios, despite there being only 6 events for the individuals with Ependymal ‘high’, 9 events for individuals with neuronal ‘high’, 5 events for individuals without radiotherapy and 12 events for individuals without gross-total resection.

The corresponding confidence intervals as well as the p values were also stable between the two models. Moreover, we calculated a multivariate Cox regression model for PFS in the PF compartment (Table S5). We observed a significant decrease in the risk of death for individuals with Ependymal biomarker ‘high’ (vs. individuals with Ependymal ‘low’), with p value <0.001. All the other effects were indicative of an increased risk of death, even though not significant. From the Kaplan-Meier estimators for the PFA-PFB-PF_SE group, we obtained a significant log rank test p value <0.001. Finally, we also calculated a multivariate Cox model for the PF-A group only also including the well-described biomarker gain of Chr1q (Table S5).

## Supplemental Information

Document S1. Figures S1–S7

Table S1. Clinical Annotation, Related to Figure 1

Table S2. Metaprogram Genes and Assignments, Related to Figures 2 and 4

Table S3. PF-EPN and ST-EPN Signatures, Related to Figures 2 and 4

Table S4. GO Term Enrichment Using Cell Population-Specific Signature and Top 30 Metaprogram Genes, Related to Figures 2 and 4

Table S5. Relative TF Activity and Metaprogram-Specific TFs, Related to Figures 2 and 4

Table S6. Cox Regression Analysis, Related to Figure 3

Table S7. DGIdb Hits and GO Enrichment, Related to Figures 3 and 5

Document S2. Article plus Supplemental Information
