# Supplementary material for: Single-Cell RNA-Seq Reveals Cellular Hierarchies and Impaired Developmental Trajectories in Pediatric Ependymoma
Source: Cancer Cell. 2020 Jul 13;38(1):44–59.e9. doi: 10.1016/j.ccell.2020.06.004 (PMC7479515; doi:10.1016/j.ccell.2020.06.004)
Supplement: Document S1. Figures S1–S7 [file mmc1.pdf]

Cancer Cell, Volume *38*

Supplemental Information

Single-Cell RNA-Seq Reveals Cellular Hierarchies and Impaired Developmental Trajectories in Pediatric Ependymoma

Johannes Gojo, Bernhard Englinger, Li Jiang, Jens M. Hübner, McKenzie L. Shaw, Olivia A. Hack, Sibylle Madlener, Dominik Kirchhofer, Ilon Liu, Jason Pyrdol, Volker Hovestadt, Emanuele Mazzola, Nathan D. Mathewson, Maria Trissal, Daniela Lötsch, Christian Dorfer, Christine Haberler, Angela Halfmann, Lisa Mayr, Andreas Peyrl, Rene Geyeregger, Benjamin Schwalm, Monica Mauermann, Kristian W. Pajtler, Till Milde, Marni E. Shore, Jack E. Geduldig, Kristine Pelton, Thomas Czech, Orr Ashenberg, Kai W. Wucherpfennig, Orit Rozenblatt-Rosen, Sanda Alexandrescu, Keith L. Ligon, Stefan M. Pfister, Aviv Regev, Irene Slavc, Walter Berger, Mario L. Suvà, Marcel Kool, and Mariella G. Filbin

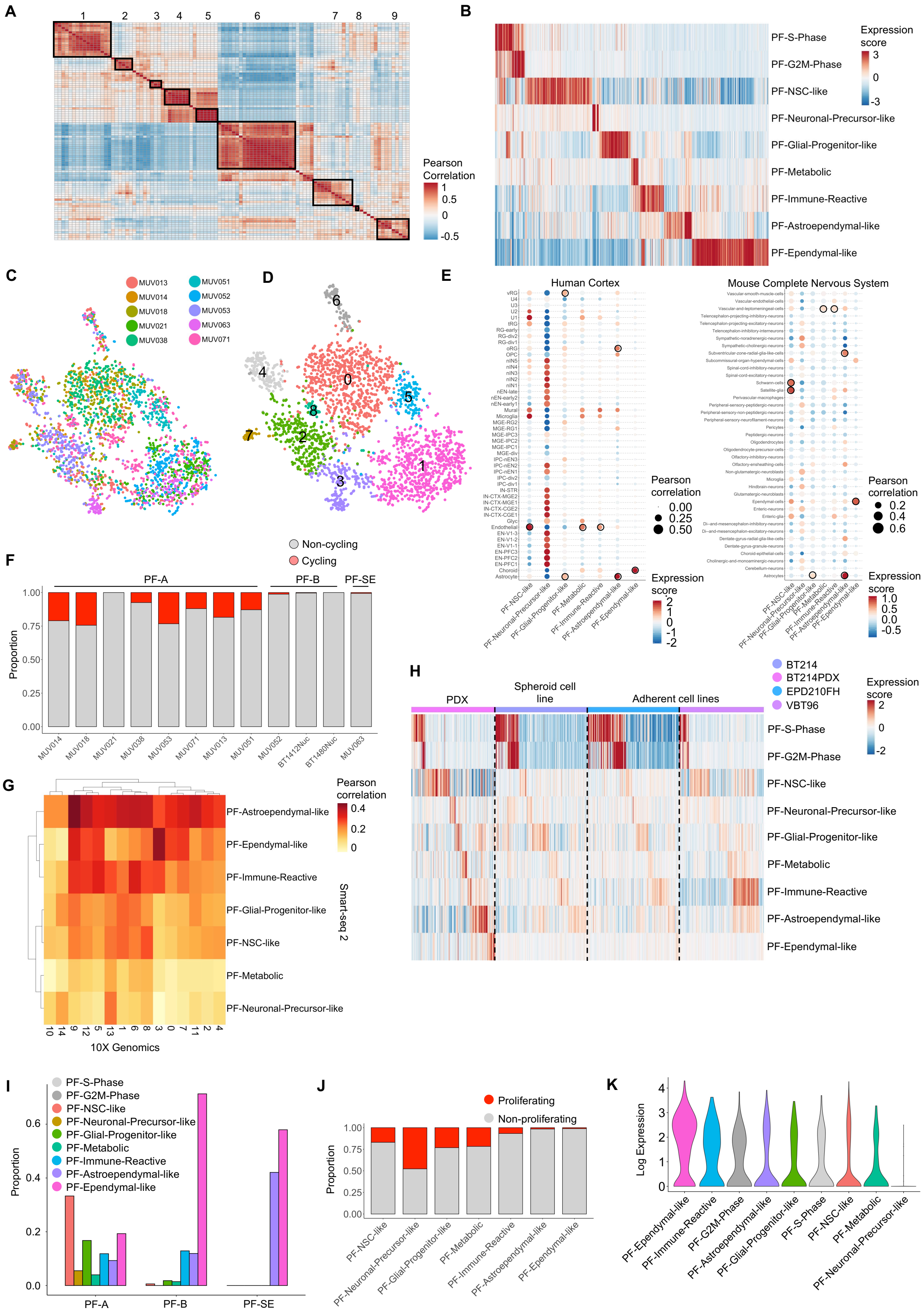

A
1 2 3 4 5 6 7 8 9
Pearson Correlation
1
0.5
0
-0.5
B
PF-S-Phase
PF-G2M-Phase
PF-NSC-like
PF-Neuronal-Precursor-like
PF-Glial-Progenitor-like
PF-Metabolic
PF-Immune-Reactive
PF-Astroependymal-like
PF-Ependymal-like
Expression score
3
0
-3
C
MUV013
MUV014
MUV018
MUV021
MUV038
MUV051
MUV052
MUV053
MUV063
MUV071
D
0 1 2 3 4 5 6 7 8
E
Human Cortex
Mouse Complete Nervous System
Pearson correlation
0.00
0.25
0.50
Pearson correlation
0.2
0.4
0.6
Expression score
2
1
0
-1
-2
Expression score
1.0
0.5
0.0
-0.5
PF-NSC-like
PF-Neuronal-Precursor-like
PF-Glial-Progenitor-like
PF-Metabolic
PF-Immune-Reactive
PF-Astroependymal-like
PF-Ependymal-like
F
Non-cycling
Cycling
PF-A
PF-B
PF-SE
Proportion
1.00
0.75
0.50
0.25
0.00
MUV014
MUV018
MUV021
MUV038
MUV053
MUV071
MUV013
MUV051
MUV052
BT1412Nuc
BT1480Nuc
MUV063
G
Pearson correlation
0.4
0.2
0
PF-Astroependymal-like
PF-Ependymal-like
PF-Immune-Reactive
PF-Glial-Progenitor-like
PF-NSC-like
PF-Metabolic
PF-Neuronal-Precursor-like
Smart-seq 2
10 14 9 12 5 13 1 6 8 3 0 7 11 2 4
10X Genomics
H
PDX
Spheroid cell line
Adherent cell lines
BT214
BT214PDX
EPD210FH
VBT96
Expression score
2
0
-2
PF-S-Phase
PF-G2M-Phase
PF-NSC-like
PF-Neuronal-Precursor-like
PF-Glial-Progenitor-like
PF-Metabolic
PF-Immune-Reactive
PF-Astroependymal-like
PF-Ependymal-like
I
PF-S-Phase
PF-G2M-Phase
PF-NSC-like
PF-Neuronal-Precursor-like
PF-Glial-Progenitor-like
PF-Metabolic
PF-Immune-Reactive
PF-Astroependymal-like
PF-Ependymal-like
Proportion
0.6
0.4
0.2
0.0
PF-A
PF-B
PF-SE
J
Proliferating
Non-proliferating
Proportion
1.00
0.75
0.50
0.25
0.00
PF-NSC-like
PF-Neuronal-Precursor-like
PF-Glial-Progenitor-like
PF-Metabolic
PF-Immune-Reactive
PF-Astroependymal-like
PF-Ependymal-like
K
Log Expression
4
3
2
1
0
PF-Ependymal-like
PF-Immune-Reactive
PF-G2M-Phase
PF-Astroependymal-like
PF-Glial-Progenitor-like
PF-S-Phase
PF-NSC-like
PF-Metabolic
PF-Neuronal-Precursor-like

Figure S1, Related to Figure 2. Characterization of metaprograms identified in PF-EPN. A. Heatmap of pairwise correlations of expression scores of NMF programs from individual PF-EPN samples, applied to all PF-EPN cells. Groups of NMF programs (black box) were merged into 9 metaprograms by hierarchical clustering (1. PF-NSC -like; 2. PF-Glial-Progenitor-like; 3. PF-Neuronal-Precursor-like; 4. PF-S-Phase; 5. PF-G2M-Phase; 6. PF-Ependymal-like; 7. PF-Astroependymal-like; 8. PF-Metabolic; 9. PF-Immune-Reactive). **B.** Gene expression score (color bar) of 2772 malignant cells (columns) for each of the PF metaprograms (row). **C.** tSNE plot of all fresh PF tumor cells, colored on the basis of individual tumor samples. **D.** tSNE plot of cells from all fresh PF-EPN samples colored by groupings from graph-based clustering. **E.** Expression score of aggregated non-malignant cell types for each PF metaprogram (color) and pairwise correlation between aggregated non-malignant cell types and EPN tumor subpopulation (circle size) in human cortex (*left*), and murine complete nervous system (*right*) reference datasets (see Methods). Black circles highlight top matches as defined by overlap of cell types between top 3 with highest expression scores and top 3 with highest pairwise correlation for each metaprogram. **F.** Relative proportion of proliferating versus non-proliferating cells per sample in all PF tumors. PF-A samples showed higher proportion of proliferating cells than PF-B and PF-SE (p value = 1.9E-21, Fisher’s exact test). **G.** Pairwise correlation of 10X Genomics snRNA-seq-derived cell subpopulations with those classified by metaprogram from the original sc/snSmart-seq2. **H.** Expression score (color bar) of cells (columns) from PF-EPN PDXs and *in vitro* cell models for PF metaprograms derived from original sc/snSmart-seq2 (rows). Side bar on the x-axis refers to samples. **I.** Relative proportion of cell subpopulation across molecular PF-EPN groups. Shannon entropy of complexity of cell subpopulation was computed for each subtype (PF-A: 1.96, PF-B: 0.26, PF-SE: 0.71). **J.** Relative proportion of proliferating versus non-proliferating cells per

subpopulation across all PF tumors. **K**. Log transformed expression of *FOXJ1* across PF-EPN metaprograms.

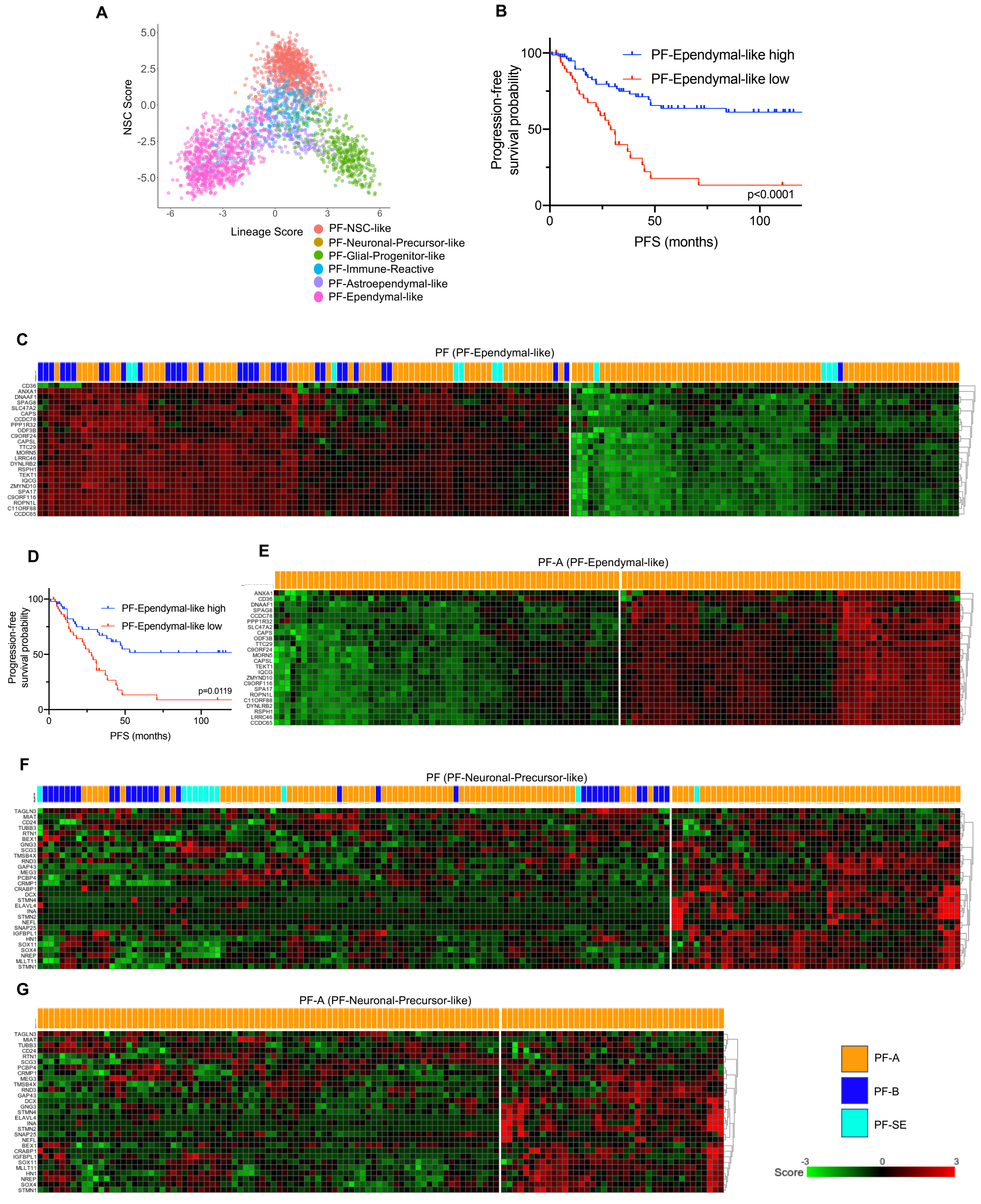

Figure S2, Related to Figure 3. Cellular hierarchies and survival implications of PF-EPN subpopulations. A. Plot of lineage- (x axis) and NSC (y axis) scores for malignant PF-EPN cells,

represented by dots. Cells are colored according to their corresponding metaprogram. **B.** PFS stratification of PF-EPN tumors according to high or low relative expression of top 30 genes for PF-Ependymal-like metaprogram in bulk RNA expression. Significance levels were determined by log-rank test. **C.** Heatmap of PF-EPN (PF-A, orange; PF-B, blue; PF-SE, cyan) bulk expression profiles clustered by K-Means on the basis of relative expression of top 30 genes for PF-Ependymal-like. **D.** PFS stratification of PF-A EPN bulk tumors according to high or low relative expression of top 30 genes for PF-Ependymal-like metaprogram. Significance levels were determined by log-rank test. **E.** Heatmap of PF-A bulk expression profiles clustered by K-means on the basis of PF-Ependymal-like metaprogram scores. **F, G.** Heatmap of PF (F) and PF-A (G) bulk expression profiles clustered by K-means on the basis of relative expression of top 30 genes for PF-Neuronal-Precursor-like metaprogram scores (PF-A, orange; PF-B, blue; PF-SE, cyan).

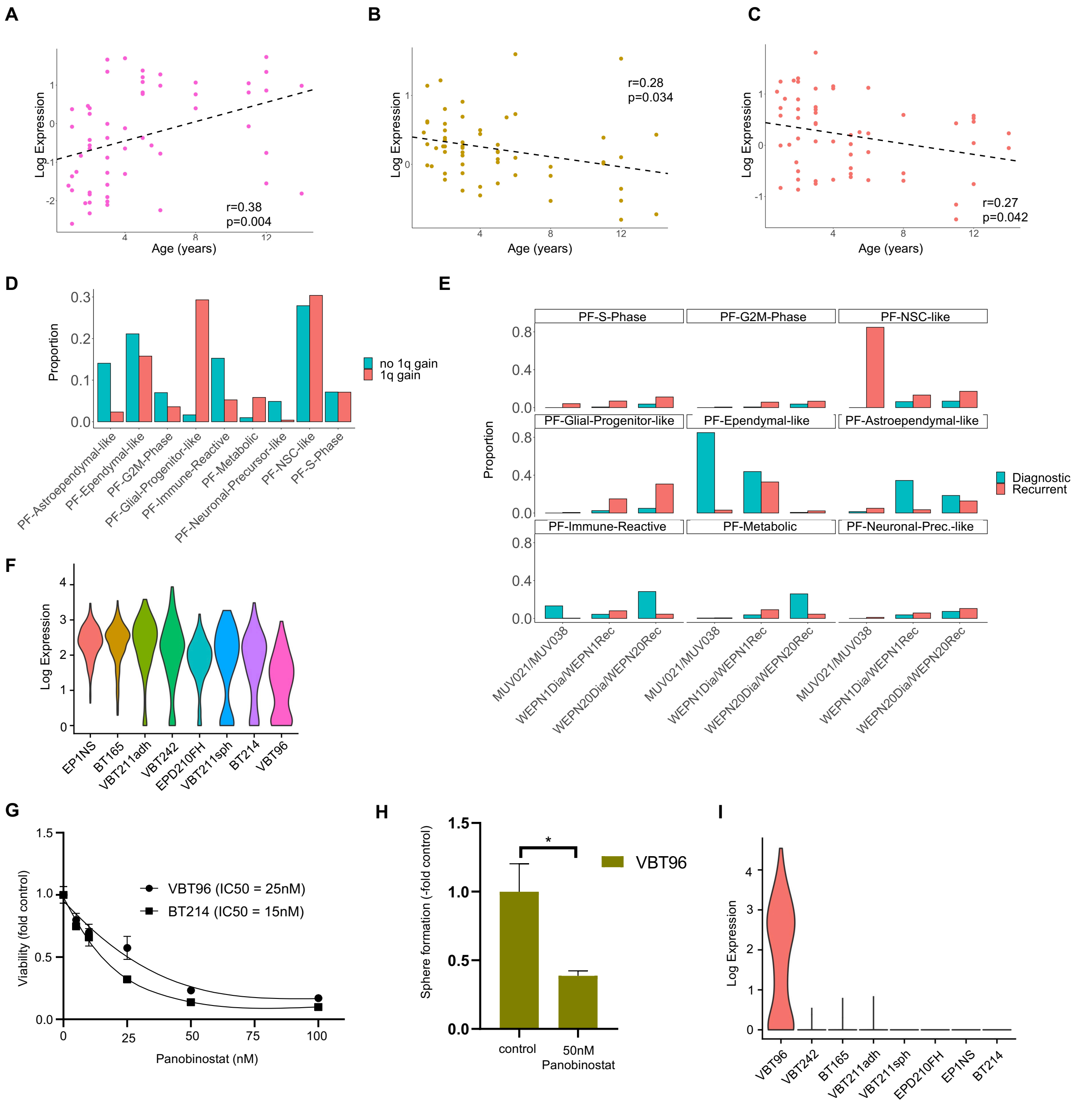

Figure S3, Related to Figure 3. Effect of Chr1q gain and tumor recurrence on cell subpopulation distribution, and targeting of subpopulation-specific vulnerabilities of PF-A. A-C. Correlation of average relative expression of top 30 genes for PF-Ependymal-like (A), PF-Neuronal-Precursor-like (B), and PF-NSC-like (C) with pediatric PF-A patient age. Best-fit regression lines, correlation coefficients and p values are shown. **D.** Relative cell subpopulation distribution between chromosome 1q-gained versus non-1q-gained PF-A specimen. **E.** Relative

proportions of cell subpopulations in matched earlier versus later recurrent PF-A samples (MUV021 versus MUV038, respectively) and matched diagnostic versus recurrent PF-A samples (WEPN1Dia vs WEPN1Rec, and WEPN20Dia vs WEPN20Rec, respectively). **F.** Log transformed expression of *HDAC2* across EPN cell models. **G.** Viability of VBT96 and BT214 cells upon 72 h treatment with increasing concentrations of the the HDAC inhibitor panobinostat was determined by CellTiter-Glo assay. **H**. Relative sphere area of the PF-EPN cell model VBT96 after 72 h treatment with the HDAC inhibitor panobinostat. Values are given normalized to the untreated control. *$p<0.05$, two-tailed student's t-test. Data are represented as mean ± SEM of triplicate values. **I**. Log transformed expression of *LGR5* across EPN cell models.

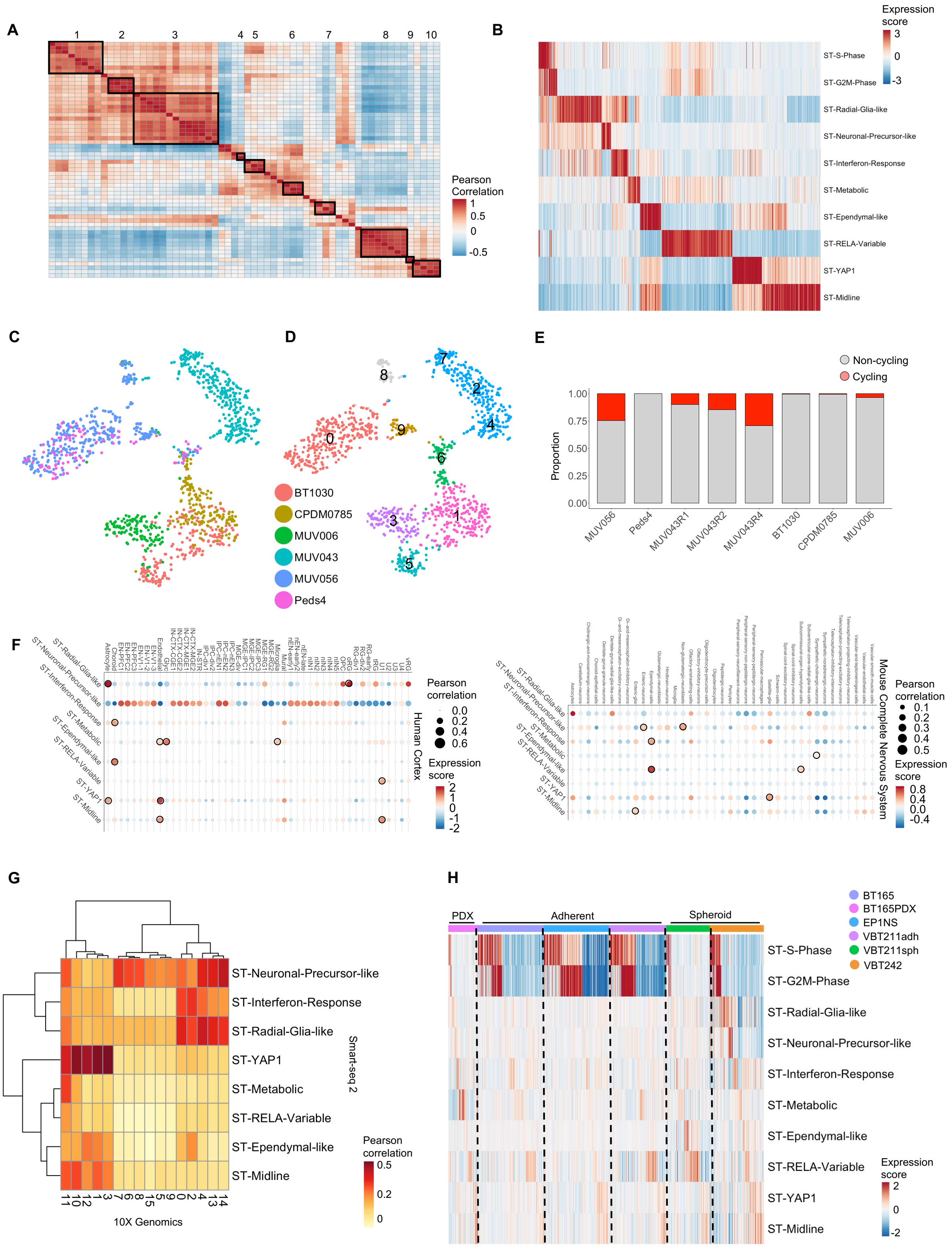
A
1
2
3
4
5
6
7
8
9
10
Pearson
Correlation
1
0.5
0
-0.5
B
Expression
score
3
0
-3
ST-S-Phase
ST-G2M-Phase
ST-Radial-Glia-like
ST-Neuronal-Precursor-like
ST-Interferon-Response
ST-Metabolic
ST-Ependymal-like
ST-RELA-Variable
ST-YAP1
ST-Midline
C
D
BT1030
CPDM0785
MUV006
MUV043
MUV056
Peds4
E
Non-cycling
Cycling
Proportion
1.00
0.75
0.50
0.25
0.00
MUV056
Peds4
MUV043R1
MUV043R2
MUV043R4
BT1030
CPDM0785
MUV006
F
Pearson
correlation
0.0
0.2
0.4
0.6
Expression
score
2
1
0
-1
-2
Human Cortex
Pearson
correlation
0.1
0.2
0.3
0.4
0.5
Expression
score
0.8
0.4
0.0
-0.4
Mouse Complete Nervous System
G
ST-Neuronal-Precursor-like
ST-Interferon-Response
ST-Radial-Glia-like
ST-YAP1
ST-Metabolic
ST-RELA-Variable
ST-Ependymal-like
ST-Midline
Smart-seq 2
Pearson
correlation
0.5
0.2
0
11
10
12
1
3
7
6
8
15
5
9
0
2
4
13
14
10X Genomics
H
PDX
Adherent
Spheroid
BT165
BT165PDX
EP1NS
VBT211adh
VBT211sph
VBT242
ST-S-Phase
ST-G2M-Phase
ST-Radial-Glia-like
ST-Neuronal-Precursor-like
ST-Interferon-Response
ST-Metabolic
ST-Ependymal-like
ST-RELA-Variable
ST-YAP1
ST-Midline
Expression
score
2
0
-2

Figure S4, Related to Figure 4. Characterization of metaprograms identified in ST-EPN. A. Heatmap of pairwise correlations of expression scores of NMF programs from individual ST-EPN samples, applied to all ST-EPN cells. Groups of NMF programs (black box) were merged into 10 metaprograms by hierarchical clustering (1. ST-YAP1; 2. ST-Ependymal-like; 3. ST-Midline; 4. ST-Neuronal-Precursor-like; 5. ST-Interferon-Response; 6. ST-Radial-Glia-like; 7. ST-Metabolic; 8. ST-RELA-Variable; 9. ST-S-Phase; 10. ST-G2M-Phase). **B.** Expression score (color bar) of 1296 malignant cells (columns) for each of the ST metaprograms (row). **C.** tSNE plot of all fresh ST tumor cells, colored on the basis of individual tumor samples. **D.** tSNE plot of cells from all fresh ST-EPN samples colored by groupings from graph-based clustering. **E.** Relative proportion of proliferating versus non-proliferating cells per sample across all ST tumors. ST-RELA samples showed higher proportion of proliferating cells than ST-YAP1 and ST-Midline (p value = 1.4E-36, Fisher's exact test). **F.** Expression score of aggregated non-malignant cell types for each ST metaprogram (color) and pairwise correlation between aggregated non-malignant cell types and EPN tumor subpopulation (circle size) in human cortex *(left*) and murine complete nervous system (*right*), reference datasets (see Methods). Black circles highlight top matches as defined by overlap of cell types between top 3 with highest expression scores and top 3 with highest pairwise correlation for each metaprogram. **G.** Pairwise correlation of 10X Genomics snRNA-seq-derived cell populations with those classified by metaprogram from the original sc/snSmart-seq2. **H.** Expression score (color bar) of cells (columns) from ST-EPN PDX and *in vitro* cell model for ST metaprogram derived from original sc/snSmart-seq2 (row). Side bar on the x-axis refers to samples.

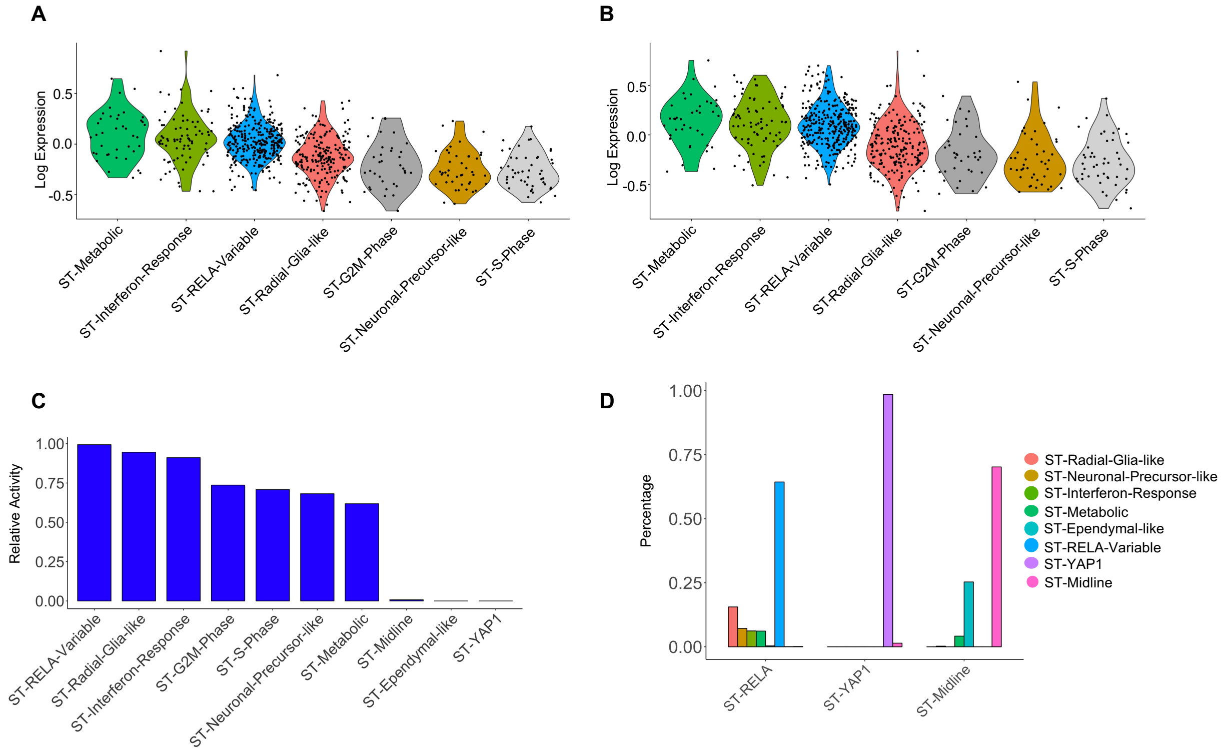

Figure S5, Related to Figure 4. Subpopulation-specific TF activities and metaprogram distribution in ST-EPN. A. Single-cell expression scores of RELA wildtype and C11orf95-RELA-regulated canonical and non-canonical NFkB target gene expression (Parker et al., 2014) across ST-EPN metaprograms. **B.** Single-cell expression scores of target genes regulated exclusively by C11orf95-RELA fusion (Parker et al., 2014) across ST-EPN metaprograms. **C.** Aggregated relative TF activity of RELA for each metaprogram inferred by SCENIC. **D.** Relative proportion of cell subpopulations across molecular ST-EPN groups. Shannon entropy of complexity of cell subpopulation was computed for each subtype (ST-RELA: 1.58, ST-YAP1: 0.15, ST-Midline: 0.76).

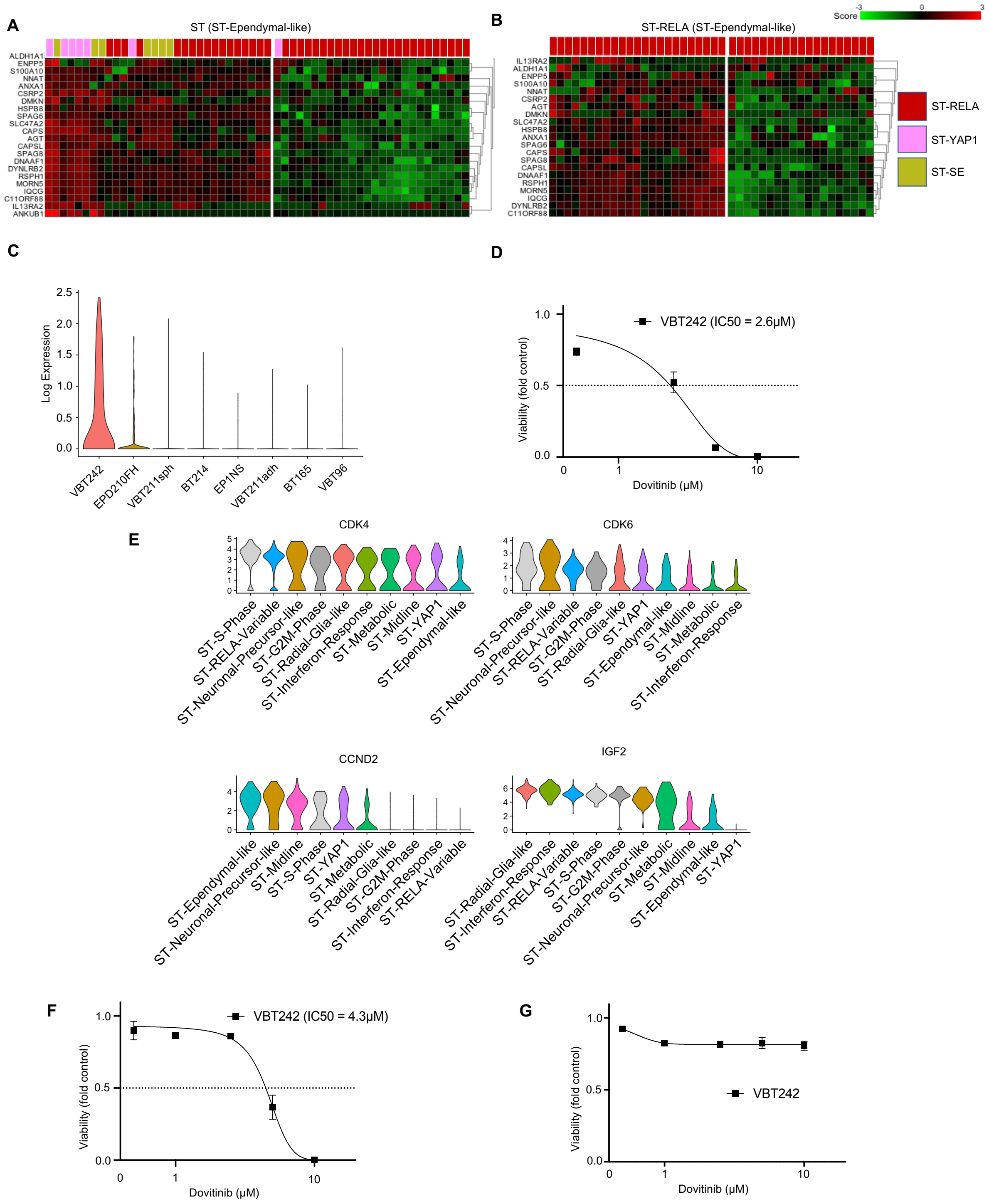

Figure S6, Related to Figure 5. Subpopulation-specific patient stratification and *in vitro* targeting of vulnerabilities of ST-EPN. A, B. Heatmap of ST-EPN (A) and ST-RELA (B) bulk expression profiles clustered by K-means on the basis of relative expression of top 30 genes for ST-Ependymal-like metaprogram (ST-RELA, red; ST-YAP1, pink; ST-SE, olive). **C.** Log

transformed expression of *FGFR3* across EPN cell models. **D.** Viability of VBT242 cells upon 72 h treatment with increasing concentrations of the FGFR inhibitor dovitinib was determined by CellTiter-Glo assay. Data are represented as mean ± SD of triplicate values. **E.** Log transformed expression of *IGF2*, *CDK4*, *CDK6*, and *CCND2*, across ST-EPN metaprograms. **F, G.** Viability of VBT242 cells upon 72 h treatment with increasing concentrations of ceritinib (F) and palbociclib (G) was determined by CellTiter-Glo assay. Data are represented as mean ± SD of triplicate values.

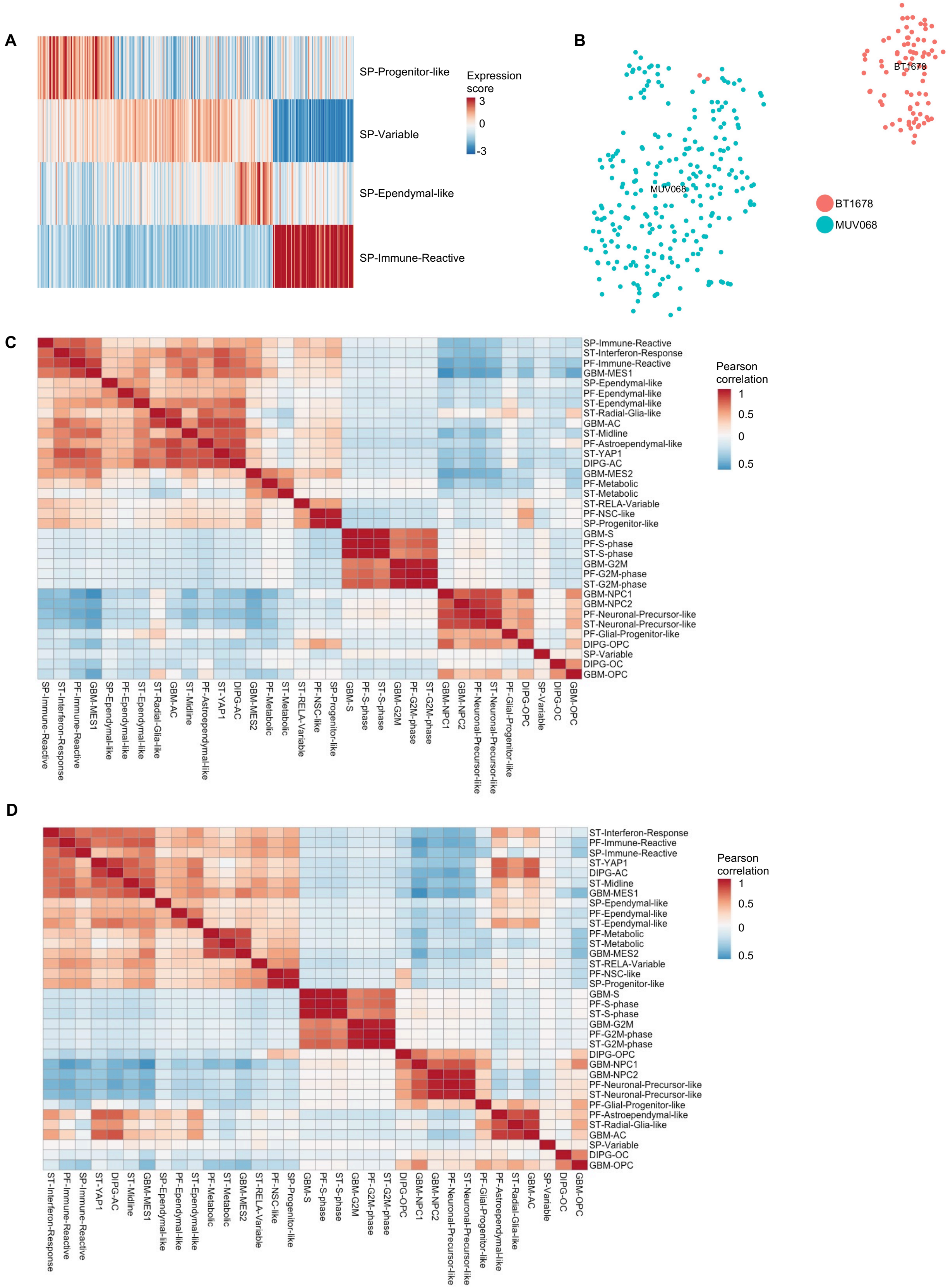

A
SP-Progenitor-like
SP-Variable
SP-Ependymal-like
SP-Immune-Reactive
Expression score
3
0
-3
B
BT1678
MUV068
BT1678
MUV068
C
SP-Immune-Reactive
ST-Interferon-Response
PF-Immune-Reactive
GBM-MES1
SP-Ependymal-like
PF-Ependymal-like
ST-Ependymal-like
ST-Radial-Glia-like
GBM-AC
ST-Midline
PF-Astroependymal-like
ST-YAP1
DIPG-AC
GBM-MES2
PF-Metabolic
ST-Metabolic
ST-RELA-Variable
PF-NSC-like
SP-Progenitor-like
GBM-S
PF-S-phase
ST-S-phase
GBM-G2M
PF-G2M-phase
ST-G2M-phase
GBM-NPC1
GBM-NPC2
PF-Neuronal-Precursor-like
ST-Neuronal-Precursor-like
PF-Glial-Progenitor-like
DIPG-OPC
SP-Variable
DIPG-OC
GBM-OPC
Pearson correlation
1
0.5
0
0.5
D
ST-Interferon-Response
PF-Immune-Reactive
SP-Immune-Reactive
ST-YAP1
DIPG-AC
ST-Midline
GBM-MES1
SP-Ependymal-like
PF-Ependymal-like
ST-Ependymal-like
PF-Metabolic
ST-Metabolic
GBM-MES2
ST-RELA-Variable
PF-NSC-like
SP-Progenitor-like
GBM-S
PF-S-phase
ST-S-phase
GBM-G2M
PF-G2M-phase
ST-G2M-phase
DIPG-OPC
GBM-NPC1
GBM-NPC2
PF-Neuronal-Precursor-like
ST-Neuronal-Precursor-like
PF-Glial-Progenitor-like
PF-Astroependymal-like
ST-Radial-Glia-like
GBM-AC
SP-Variable
DIPG-OC
GBM-OPC
Pearson correlation
1
0.5
0
0.5

Figure S7, Related to Figures 6 and 7. MPE metaprogram characterization and correlation of metaprograms within EPN and across other glioma types. A. Expression score (color bar) of 333 malignant cells (columns) for each of the SP metaprograms (row). **B.** tSNE plot of all MPE tumor cells, colored on the basis of individual tumor samples. **C, D.** Pairwise correlation of expression score of metaprograms defined in EPN, DIPG, and GBM and applied to cells from DIPG (C) and GBM (D) datasets.
